# Metallic Glass Nanoparticles Synthesized via Flash Joule Heating

**DOI:** 10.1021/acsnano.5c02173

**Published:** 2025-05-15

**Authors:** Hang Wang, Nathan Makowski, Yuanyuan Ma, Xue Fan, Stephen A. Maclean, Jason Lipton, Juan Meng, Jason A. Röhr, Mo Li, André D. Taylor

**Affiliations:** † Department of Chemical and Biomolecular Engineering, Tandon School of Engineering, 5894 New York University, New York, New York 11201, United States; ‡ College of Materials, Shanghai Dianji University, Shanghai 201306, China; § School of Material Science and Engineering, 1372 Georgia Institute of Technology, Atlanta, Georgia 30332, United States

**Keywords:** metallic glass, nanoparticles, flash Joule heating, metastable phase, alloy nanoparticles, ternary alloys, ternary phase diagram

## Abstract

Metallic glass (MG) nanoparticles have attracted intensive research interest for their promising mechanical and catalytic applications. However, current production methods lack the ability to precisely control phase, composition, and morphology, making it challenging to explicitly study their structure–property relationship. Here, we report a direct one-step synthesis of MG nanoparticles using flash Joule heating (FJH) that allows us to produce nanoparticles with desired phase, composition, and morphology. With the fast and controllable cooling attainable through FJH, we can produce fully amorphous Pd–P, Pd–Ni–P, and Pd–Cu–P nanoparticles with precise control in alloy composition and particle size (2.33 nm ± 0.83 nm). As a demonstration of potential application, we show the improved oxygen evolution activity (∼300 mV lower onset potential) of the MG nanoparticles over their crystalline counterparts and long-term stability in 60-h testing.

## Graphical abstract

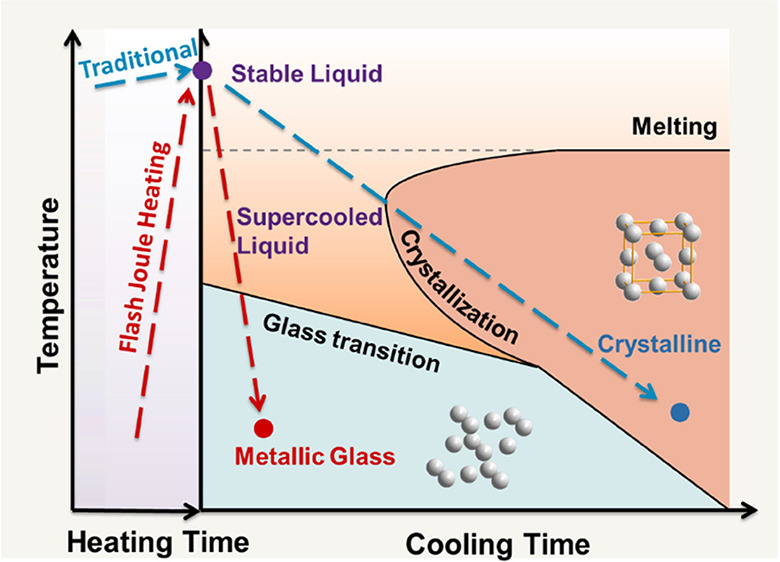

## 1 Introduction

Glasses are solids with an atomic arrangement that lacks long-range order when maintained below their glass transition temperatures.^1^ Specifically, they possess a liquid-like disordered structure while retaining stiffness and hardness.^2^ Among the wide range of glassy materials, metallic glasses (MG) stand out as a special type primarily composed of metallic elements. They combine the physical properties of glasses (brittle and flowing) with those of metals (stiff and tough). Some MG alloys are three times stronger than titanium and are extremely lightweight.^3^ Due to an abundance of uncoordinated surface sites that can serve as catalytic reaction sites, MGs can have high catalytic activity and long-term stability as compared to their crystalline counterparts.^2,4–7^ Despite their enormous potential to be used in a wide range of applications, current synthesis methods cannot deliver nanosized MGs of diverse compositions with precise control of morphology and size.

Traditional top-down MG synthesis methods typically achieve nanoscale morphology from the dealloying,^4^ corrosion,^5^ or templating^6,7^ of bulk MG. Though these techniques are relatively simple, the preparation of bulk MG precursors involves quenching liquids or electro-sputtering, which makes the entire process time-consuming and complex. A few bottom-up synthesis methods, including pulse electrodeposition,^8^ solution chemical synthesis,^9^ and inert gas condensation,^10,11^ have been used in recent years that can give certain degrees of control over phase-composition and morphology. However, each approach has its own limitations, which range from a narrow alloy composition space^8^ to an inability to obtain a uniform amorphous phase,^9^ or achieve a desired morphology.^10,11^ Moreover, all these techniques require multistep processing, which is costly, time-consuming, and error-prone. In order to realize MG nanoparticle catalysts, an effective and facile synthesis methodology with precise control over phase, composition, and morphology is needed.

Flash Joule heating (FJH) is a convenient selective-area heating technique that has been used for the production of bulk MGs^12–14^ and other functional materials including graphene,^15^ carbides^16^ and 2-dimensional MoS_2._ ^17^ Different from conventional heating processes where samples are passively heated up through convection and conduction (Figure 1a), FJH is based on heat generated from an electrical current passing through a sample (Figure 1b). Only the selected region through which current passes produces heat, while the surrounding environment remains at ambient temperature. The large temperature gradient between the Joule heated sample and the environment can lead to ultrafast cooling. If the cooling rate is fast enough, glass formation can be readily achieved sans crystallization (Figure 1c). Using this principle, FJH has initially been used as a feasible method for molding bulk Cr–Zr alloys,^12^ with later explorations focusing on mechanical manufacturing applications for other bulky Cu-and Ni-based MG alloys.^13,14,18^ Recently, FJH was applied in the synthesis of high–entropy alloy (HEA), oxide and carbide nanoparticles.^19–21^ Due to the high mixing entropy and thermal stability, nanoparticles made of HEAs were produced using FJH. However, it is much more demanding to use FJH to produce MG nanoparticles: The first challenge is the faster critical cooling rate required for MGs, especially binary and ternary alloys widely used for catalysts. The second is the proper selection of precursors that can form nanoparticles, due largely to diverse melting temperatures among very different types of pure metals. The third is the complicated process of mixing, alloying, and glass formation (or avoidance of crystallization) in the environment unique to FJH.

**Figure 1:**
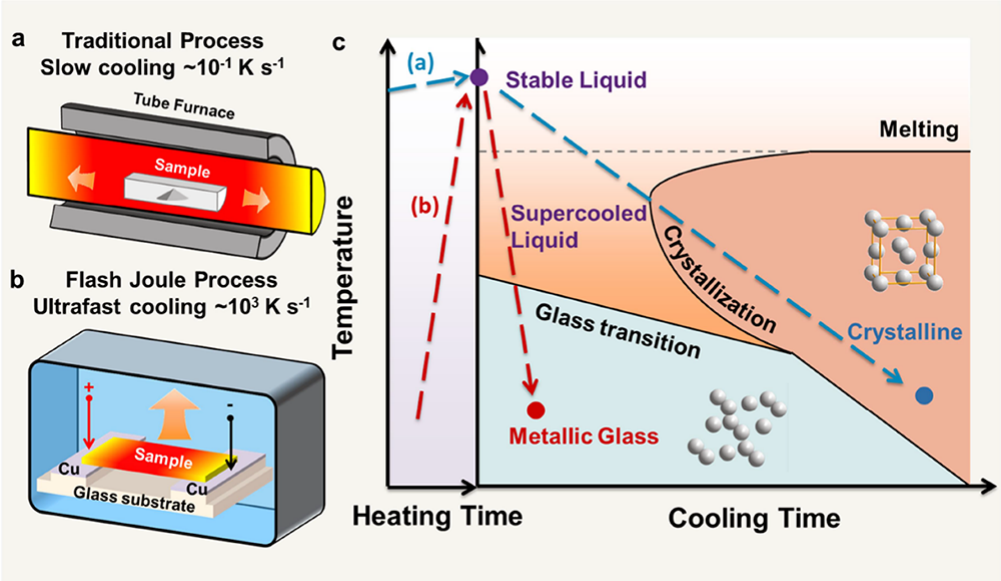
FJH processing and phase transformation with crystallization or glass formation during cooling. Schematics for (a) a traditional heating–cooling process. The cooling rate for a conventional laboratory tube furnace is ∼0.1 K s^–1^ and (b) the FJH process with a cooling rate of ∼1000 K s^–1^. (c) Illustration of temperature vs time diagram of supercooled liquid alloys and glass transformation. Red and blue arrows represent the thermal profiles of the traditional heating in (a) and FJH processes in (b), respectively.

Herein we report a one-step direct synthesis of fully amorphous, compositionally diverse, and narrowly size-distributed MG nanoparticles. The high holding temperature (1173 K) during this process guarantees precursor decomposition, particle melting, and elemental mixing. The subsequent fast cooling rate (∼1000 K s^–1^) surpasses the critical cooling rate for crystallization (∼1 K s^–1^)^22^ for metal phosphide alloys (Pd–P, Pd–Cu–P and Pd–Ni–P). Using this technique, we show that not only can MG nanoparticles be made but also that the MG composition space can be significantly expanded beyond the current feasible range using conventional synthesis techniques. Previously, other groups have synthesized metallic glasses through flash carbothermic reaction (FCR).^23^ Expanding upon their discoveries, we demonstrate the ability to rigorously control the particle size by changing the alloy composition and the parameters in FJH. Using our approaches, we demonstrate that the nanoparticle size can be controlled with an average diameter ranging from 2.33 nm ± 0.83 nm (coefficient of determination, *R* ^2^ = 0.978) to 36.5 nm ± 10.1 nm (*R* ^2^ = 0.98) by optimizing the thermal profile, precursor combination, and substrate. Our method of producing particles leaves them suspended on a carbon fiber substrate, allowing for easy implementation into various applications like catalysis. The as-synthesized MG nanoparticles show outstanding oxygen evolution reaction stability (>60 h) as compared to their crystalline counterparts (<1 h). We suggest that this FJH method could enable broader applications such as alloy microstructure engineering^24^ and steel manufacturing,^25^ along with the formation of thermoplastic elastomers,^26^ colloids,^27^ and biomaterials.^28^

## 2 Results and Discussion

### 2.1 FJH and Fast Cooling for Glass Formation

The MG synthesis was performed using a homemade FJH system in an Ar glovebox (Figure S1). This approach differs from the traditional method of melting or mixing metals using a crucible and furnace (Figure 1a), as it utilizes a substrate comprising of a carbon fiber strip (60 mm × 2 mm) connected to two electrodes (Figure 1b). The mixed precursor solution is loaded by drop-casting onto the central (20 mm × 2 mm) region of the carbon fiber strip (Figure 2a). For a standard FJH operation, 15 V is applied to the substrate (*R*_o_ = 12 Ω; *I* = 1.25 A) for 2 s followed by an immediate shut down of the power supply, which allows the system to cool down without assistance. We illustrate the visible color change and irradiation of the carbon substrate undergoing Joule heating, shown in Figure 2a. A high-speed infrared camera with a time-resolution of 10 ms documented the complete heating and cooling process of the sample during FJH (Video S1).

**Figure 2:**
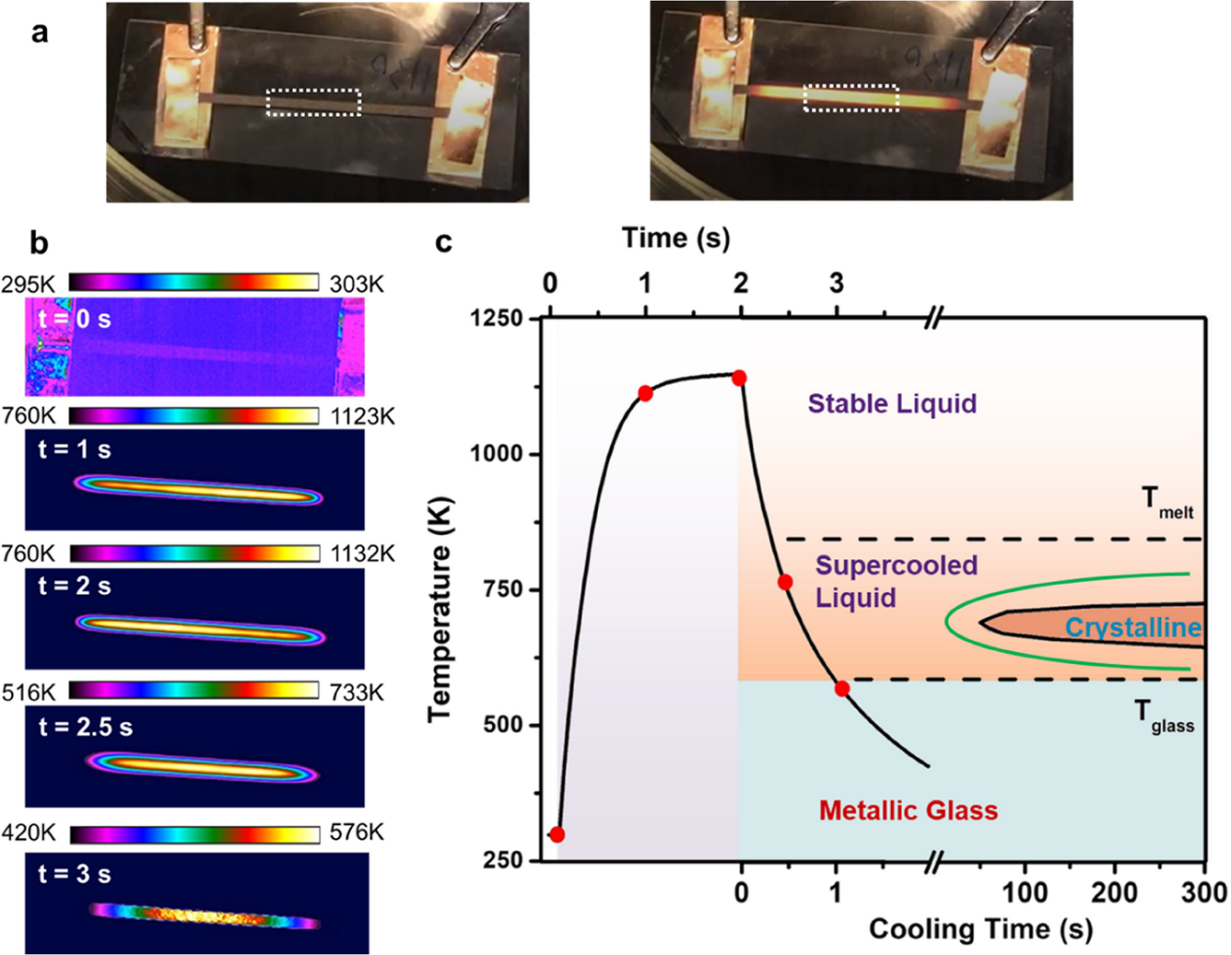
Thermographic analysis of the FJH process. (a) Photographs of the substrate before and during the FJH process. The dashed white box marks the precursor loading region. (b) Thermographic profile images of the substrate during the FJH process at *t* = 0, 1, 2, 2.5 and 3 s on the thermal profile. (c) Thermal profile of the FJH process as measured by thermographic imaging. The experimental TTT for Pd_40_Ni_10_Cu_30_P_20_ from Loffler et al.^22^ and the estimated TTT for Pd–Ni–P are shown in indigo and green curves.

To quantitatively study the temperature distribution and thermal profile during the FJH process, a thermographic imaging analysis was conducted (Video S2). We show the temperature contour map at different times in Figure 2b. The maximum temperature (*T*_max_) measured among all spots on the substrate was ∼1132 K with a temperature variation of ∼70 K in the precursor loading region. Since this temperature variation is negligible as compared to *T*_max_, we assume that the thermal profile at any spot on the heated region is approximately uniform.

The thermal profile, calculated from thermographic imaging, is plotted in Figure 2c together with the time–temperature-transformation (TTT) diagram of Pd_40_Ni_10_Cu_30_P_20_. The ramping and cooling profile shows the exponential increase and decay, as expected from Newtown’s Law of cooling (Figure S2). The maximum temperature of 1100 K exceeds the precursor melting temperature of 900 K, enabling complete melting. Holding the voltage for 2 s ensures adequate elemental mixing during the FJH process. The average ramping rate from voltage onset was 2505 K s^–1^ and the average cooling rate immediately following voltage shutoff was 1485 K s^–1^ (Table S1). Although previous FJH literature usually only reports the initial cooling rate,^12,19^ the average cooling rate from the melting point (*T*_melt_) to the glass transition point (*T*_g_) is the critical parameter for glass formation, as it more appropriately describes the solidification process. In order to form a glass, the liquid formed above the liquidus temperature must be cooled sufficiently fast to avoid crystallization (Figure 1c). In our case, the average cooling rate from *T*_melt_ (900 K) to *T*_g_ (600 K) was 426 K s^–1^ (Table S1). In comparison, previous literature has found that a cooling rate of 1 K s^–1^ is required to obtain a multicomponent Pd–Ni–Cu-P glass.^22^ As shown in Figure 1c, the fast cooling rate during FJH is far faster than the critical cooling rate to form this alloy composition. Extrapolating this, we expect that FJH will allow us to obtain glasses with binary or ternary compositions that require higher cooling rates. This unique aspect of our FJH allows us to synthesize MG nanoparticles that have been difficult, if not impossible, before.

As proof-of-concept, we first demonstrate the synthesis of binary Pd–P MG nanoparticles through a rapid 2s FJH process, utilizing PdCl_2_ and PPh_3_ as precursors. Additional details regarding the precursor selection, loading, and the FJH setup can be found in the Supporting Information. Scanning electron microscopy (SEM) energy dispersive X-ray analysis (EDX) (Figures S3 and S4) confirmed that the composition of the synthesized nanoparticles is Pd_75_P_25_ (Pd_3_P), the Pd–P compound with the highest melting point and thermal stability.^29^ X-ray diffraction (XRD) patterns were used to compare Pd–P samples synthesized under increasing heating durations (0.5, 2, and 5 s) (Figure 3a). We observe the absence of a diffraction peak at 2θ = 40° for the MG nanoparticles processed by heating for 2 and 5 s, indicating a high degree of disorder.^30^ Selected area electron diffraction (SAED) analysis on one of the synthesized nanoparticles reveals a broad diffraction ring (Figure 3b), consistent with prior observations of an amorphous phase.^31^ Scanning transmission electron microscopy (STEM) EDX mapping shows that the synthesized particles have a homogeneous composition with no observable phase separation between constituent elements (Figure 3c).

**Figure 3:**
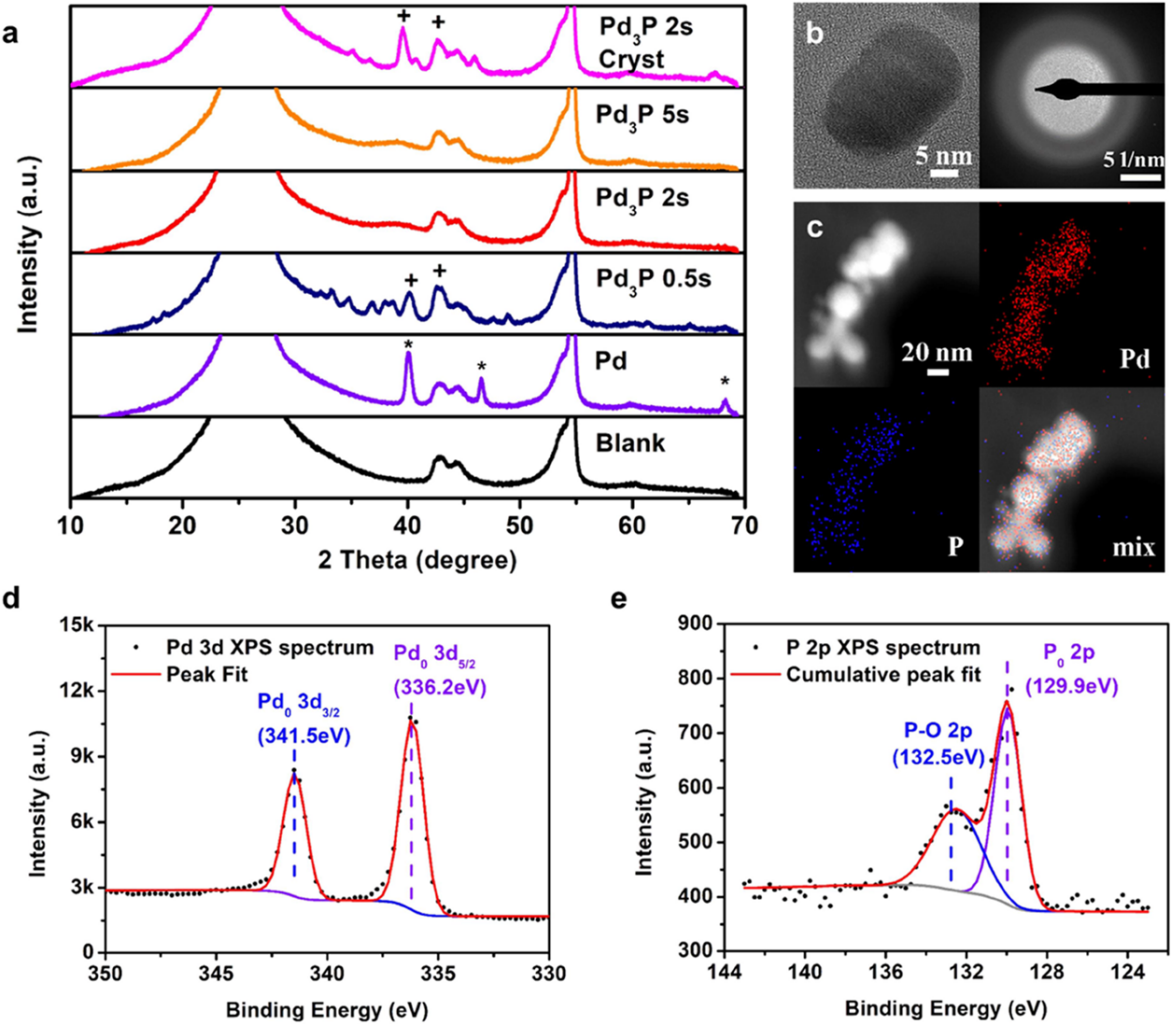
Amorphous MG nanoparticles synthesized by FJH. (a) XRD patterns obtained from binary MG nanoparticles prepared by various flashing times. The crystalline (“cryst”) sample was heated at 1073 K for 2 h in a tube furnace to allow for crystallization. (b) TEM image and corresponding SAED pattern of a Pd_3_P nanoparticle, (c) STEM-EDX mapping of amorphous Pd_3_P nanoparticles. (d, e) High-resolution XPS spectra and corresponding cumulative fittings of Pd 3d and P 3p peaks for amorphous Pd_3_P.

From X-ray photoelectron spectroscopy (XPS) spectra, we show that both Pd and P atoms in the alloys are in a metallic state (Figure 3d,e).^32^ The binding energy of Pd 3d_5/2_ is 336.2 eV, matching that of pure Pd metal. The main peak of P 2p, located at 129.9 eV, undergoes a slight negative shift of 0.3 eV compared to pure P, indicating an electron donation from Pd to P. This is expected for metallic Pd–P alloys.^33^ Since no Pd 3d side peak was detected, the side peak of P 2p at 132.5 eV is assigned to the surface peak (exposure to air) rather than the presence of phosphates or polyphosphates, which is very common in phosphide nanoparticles.^34–36^ The surface peak also suggests the existence of P-rich surface shell of the nanoparticle.^37^

Remarkably, we reveal that XRD diffraction peak intensity at 2θ = 40° decreases with increasing flashing time (the time during which the voltage is applied), indicating a lower degree of particle crystallinity (Figure 3a). In traditional thermal processes (Figure 1a), a longer heating period will lead to increased crystallization, as disordered structures have sufficient time to form structural order.^38,39^ By contrast, longer heating times during FJH led to decreased crystallinity, as amorphous Pd_3_P nanoparticles were obtained when heated for 2s or longer. This can be explained by the following mechanism: (1) The direct decomposition product of the PdCl_2_ and PPh_3_ precursors are crystalline; (2) short flashing times of 500 ms lead to a lower absolute temperature and insufficient time for melting (Figure 2c), which leads to crystalline domains; (3) when applying a longer heating time (≥2 s), the alloy particles are fully melted allowing for the formation of an amorphous phase during the cooling process. Increasing the flashing duration to 5 s or longer does not yield crystalline Pd_3_P, supporting the proposed mechanism.

To prove that the glass formation is due to the rapid cooling rate afforded by the FJH process, the FJH-synthesized MG nanoparticles were subsequently heated to 1073 K for 2 h in a tube furnace followed by cooling at a rate of ∼0.1 K s^–1^ to simulate a standard thermal annealing. A prominent diffraction peak at 40 ° was observed, suggesting that the previously amorphous nanoparticles had recrystallized (Figure 3a). Although the heating temperature (1073 K) is lower than the melting point of bulk Pd_3_P (1320 K) or other Pd–P compounds,^29^ crystallization still occurs due to the time–temperature equivalence principle exhibited in the TTT diagram.^40^ By supplying energy to the kinetically trapped amorphous particles for a long enough time, they are able to reorient themselves into a crystalline lattice. Compared with previously reported Pd_3_P that was completely^33^ or partially crystalline,^41^ we confirm the formation of fully amorphous Pd_3_P nanoparticles. This indicates that our FJH enables the formation of glassy alloys (e.g., Pd_3_P) that have not been previously documented in the existing literature.

### 2.2 Alloy Composition Control

To demonstrate the capability of FJH to control the composition of MG nanoparticles, we select binary (Pd–P) and ternary (Pd–Ni–P and Pd–Cu–P) alloy systems as examples, considering the significant research interest and reported catalytic performance on these materials.^4–7^ For the Pd–P binary system, FJH can be used to prepare Pd_100–*x*_P_*x*_ alloys where *x* < 25. When the P precursor (triphenyl phosphine, PPh_3_) is loaded in excess, Pd_3_P forms when the flashing time is longer than 500 ms (Figures 3a and S5). Considering that PPh_3_ fully sublimates when heated by itself (Figure S6), we attribute the formation of the Pd–P binary alloy to the affinity of Pd to P, which immobilizes the vaporous P. The selectivity of Pd_3_P regardless of Pd to P precursor ratio indicates that Pd_3_P has superior thermal stability during the FJH process (*T*_max_ = 1100 K) as compared with all other higher P % alloys. We suspect that even though an excess of P was used, the melted Pd–P alloy droplets release gaseous phosphorus compounds and decompose to Pd_3_P.^29^ When the phosphorus precursor was insufficiently loaded (P/Pd molar ratio <1:3), the reaction led to the formation of new complex phases of Pd–P alloys, including potential phases such as Pd_7_P_2_ and pure Pd (Figures S5 and S7).

By adding nickel salt (NiCl_2_) to the precursor mixture, we can make a series of Pd–Ni–P ternary alloys. STEM-EDX mapping demonstrates uniform mixing and elemental distribution (Figure S8). As listed in Table S2, the final product of PdCl_2_:NiCl_2_:PPh_3_ (1:1:3 molar ratios) results in the formation of Pd_61_Ni_20_P_19_. We suggest that the deviation between the precursor and final product molar ratio, which has been reported by previous FJH studies,^19,20^ originates from the sublimation of NiCl_2_. As will be discussed in Section 2.3, this can be solved by optimizing the recipe. The XPS binding energies of Pd 3d_5/2_, Ni 2p_3/2,_ and P 3p_3/2_ for as-synthesized Pd_60_Ni_20_P_20_ were 335.9, 852.6, and 130.0 eV, respectively (Figure S9). Although the valence states of Pd, Ni, and P are all metallic, The coupling of a negative shift of the Pd 3d_5/2_ peak with a positive shift in the Ni 2p_3/2_ peak indicates a charge transfer from Ni to Pd.^42,43^ The low-crystallinity of Pd–Ni–P ternary alloys is confirmed by the missing diffraction peak at 2θ = 40° in the XRD patterns (Figure S10). Note that under the same FJH parameters, crystalline binary Pd_3_P and PdNi particles were formed. Such an amorphous phase can be maintained when the loading is increased from 5 to 10 or 15 μmol/cm^2^ (Figure S11), though particle aggregation occurs (Figure S12). This suggests that the addition of nickel improves glass formation ability, which is also verified by molecular dynamic simulation. The TTT diagrams of various Pd–Ni binary alloys were simulated by molecular dynamic calculation to determine the critical cooling rate of the alloy systems (Methods, Figure S13 and S14). The extremely high critical cooling rates for Pd–Ni alloys (10^11^–10^13^ K s^–1^) compared to Pd_3_P suggest that the improved glass formability of Pd–Ni–P alloys comes from metal phosphorization rather than elemental mixing.^44^

We plot the compositions of synthesized Pd–Ni–P alloys (see details in Table S3) as red triangles in the ternary phase diagram (Figure 4a) and show that the final Pd to Ni ratios (1.87, 1.0, 0.68) adhere very closely to the designed values (2, 1, 0.5). By comparing the conventional glass formation region (black)^45,46^ with our reported FJH glass formation region (red), we reveal a significantly expanded MG composition space. Interestingly, all products follow the formula (Pd_3_P)_*x*_Ni_100–4*x*_ (Figure 4a), which are essentially binary alloys between Pd_3_P and Ni. Following the melting-fusion mechanism previously outlined in literature, we explain the process of alloy formation during FJH by the proposed mechanism in Figure 4b.^20^ While drop-casting the precursor solution onto the CFP promotes effective mixing of the precursors, the higher oxidative potential of Pd­(II) compared to Ni­(II) results in the reduction of Pd­(II) to Pd_3_P at a lower temperature.^47,48^ Because of the immiscibility and thermal instability of Ni–P alloys, the Ni­(II) is reduced to P-doped nickel (Ni_98_P_2_) with the addition of the P precursor, rather than high P% compounds like Ni_2_P (Figure S15). We suggest that the Pd_3_P and Ni phases form separately at different temperatures (Figure 4b-iii). As *T*_max_ (1183 K) exceeds the formation temperature of both Pd_3_P and Ni phases,^47,48^ the particles subsequently melt and fuse together (Figure 4b-iv). The fused alloys remain stable during the FJH process, resulting in the retention of the (Pd_3_P)_*x*_Ni_100–4*x*_ composition after heating and cooling (Figure 4b-v). Due to the fast cooling rate, the supercooled nanodroplets solidify into metallic glass nanoparticles (Figure 4b-vi).

**Figure 4:**
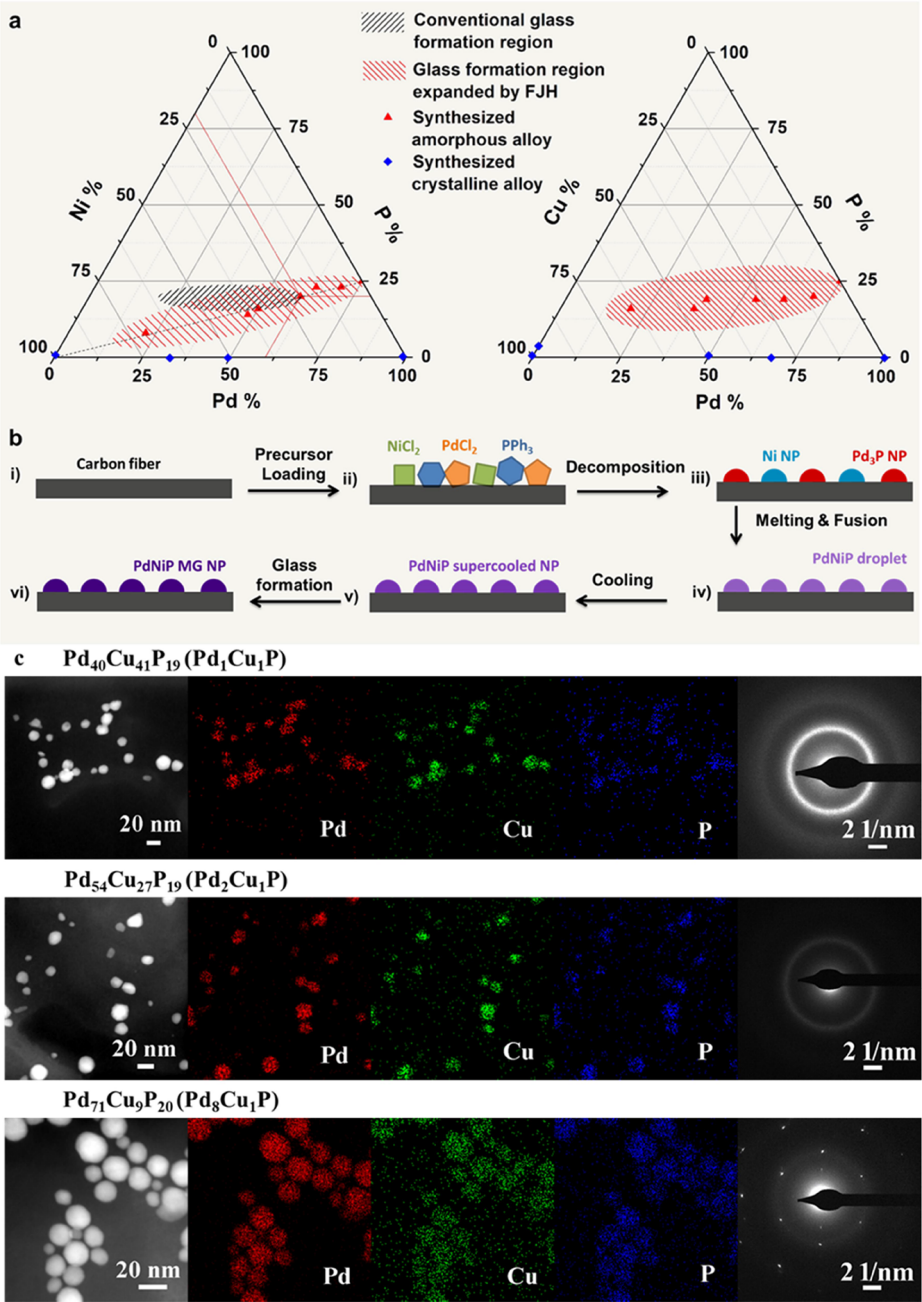
Ternary Pd–Ni–P and Pd–Cu–P alloy composition control. (a) Ternary phase diagrams. The crystalline and amorphous materials are labeled with blue diamond and red triangles, respectively. The black region denotes the conventional glass formation region, and the red region denotes the FJH expanded glass formation region.^45,46^ (b) Proposed mechanism for Pd–Ni–P alloy formation. (c) STEM-EDX maps and SAED patterns of various Pd–Cu–P MG nanoparticles (Pd_40_Cu_41_P_19_, Pd_54_Cu_27_P_19_ and Pd_71_Cu_9_P_20_).

To further test the versatility of FJH and the underlying mechanisms observed, we added Cu to Pd–P system to show that ternary Pd–Cu–P amorphous alloys can also be formed. The XPS binding energies of Pd 3d_5/2_, Cu 2p_3/2,_ and P 3p_3/2_ at 336.1, 932.1, and 130.2 eV, respectively, confirm the metallic valence states (Figure S16). We confirm the amorphous state of the Pd–Cu–P alloy nanoparticles using XRD, but note that the crystallinity increases with the Pd % based on the increased intensity of the XRD peak at 2θ = 40° (Figure S17). The appearance of diffraction spots, as well as the decreasing intensity of the diffuse diffraction ring in the SAED patterns of the Pd_1_Cu_3_P, Pd_1_Cu_1_P, and Pd_3_Cu_1_P alloys provide further evidence that a higher ratio of Pd:Cu leads to a more crystalline product (Figure 4c). In fact, both XRD and SAED imply a crystalline Pd–Cu phase separation. Moreover, we reveal that the influence of Cu alloying is not entirely similar to that of Ni, reflected in the observation that the composition of Pd–Cu–P alloys do not follow a formula similar to that of the Pd–Ni–P ternary alloys. By comparison, the Pd–Cu–P system retains a relatively high P % even when Pd % is low (Figure 4a). This can be explained by the fact that a reduction of Cu­(II) to Cu(0) occurs at a lower temperature (∼1000 K) than that of Ni­(II) to Ni(0) (∼1200 K), which is closer to the Pd_3_P formation (950 K) temperature.^47^ The synchronous reactions of Cu reduction and Pd_3_P formation enable direct alloying and a higher phosphorus solubility.

### 2.3 Particle Purity, Size, and Distribution Control

Morphology and size control are key factors to be considered when judging a methodology for nanomaterial synthesis, especially for catalytic applications.^21^ In our FJH, the effects due to precursor selection, thermal profile, and alloy composition were systematically evaluated to determine their effects on particle size and composition. A desired particle size distribution can be obtained by optimizing the FJH thermal profile and recipe without sacrificing phase and composition control (Figure 5a–c). In general, uniform precursor loading, the choice of inorganic anion precursors, and the mixing of multiple elements facilitates a reduction in particle diameter.

**Figure 5:**
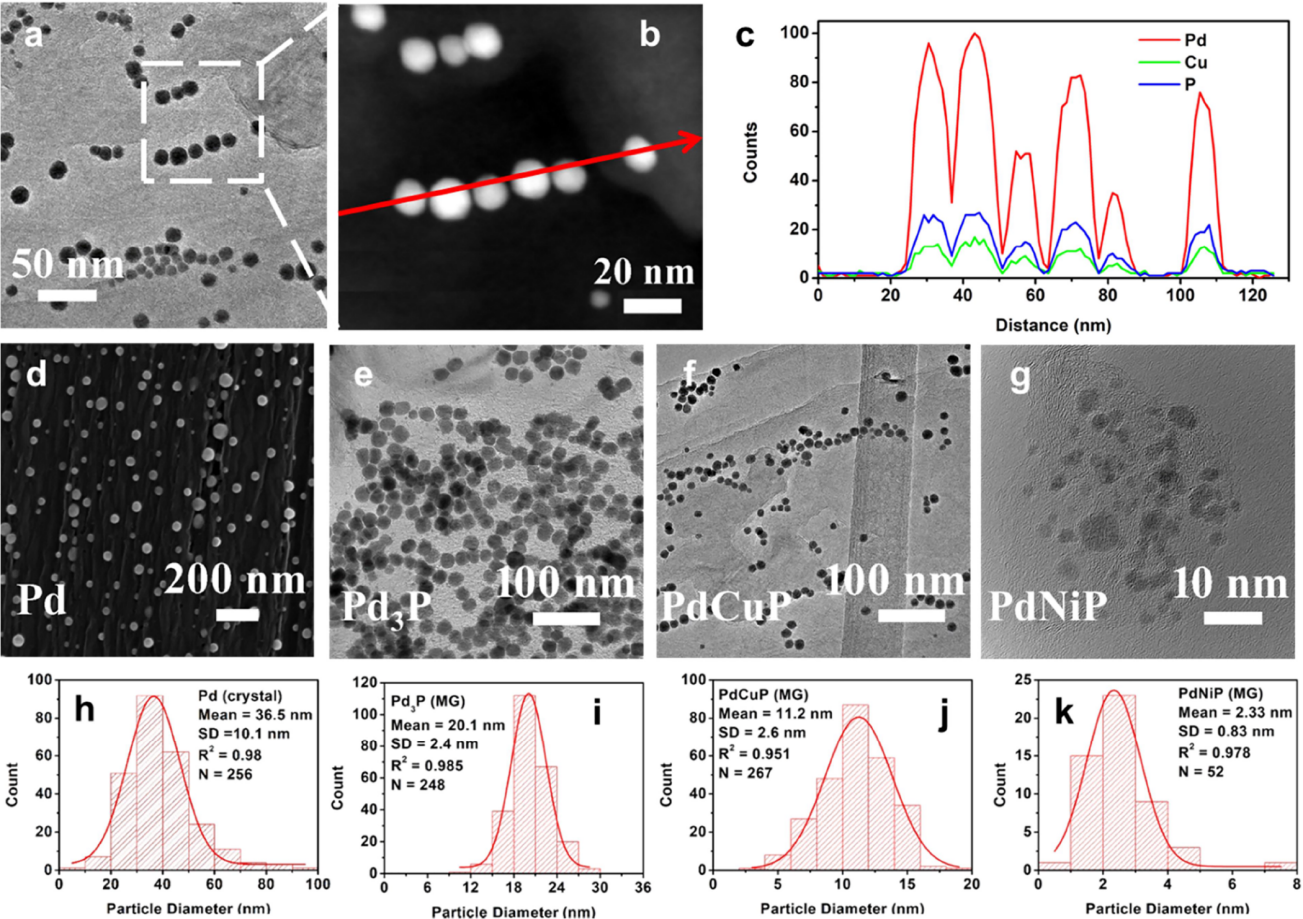
Particle size distribution control. (a) STEM image of Pd–Cu–P alloy particles on a carbon fiber, marked with a dashed box showing the location of the subsequent TEM and EDX line scan. (b) TEM image. The red arrow shows the direction of the EDX line scan. (c) EDX line scan of six Pd–Cu–P particles. (d) SEM image of pure Pd nanoparticles. (e) TEM image of amorphous Pd_3_P nanoparticles. (f) TEM image of amorphous Pd–Cu–P nanoparticles. (g) TEM image of amorphous Pd–Ni–P nanoparticles. (h–k) shows the corresponding particle size distributions. “MG” and “crystal” labels refer to metallic glass and crystalline phases, respectively.

A uniform precursor loading is a precondition toward achieving a good particle formation. When applying the aqueous precursor solution (0.1 M NiCl_2_ in H_2_O, Figure S18) to the hydrophobic carbon fiber substrate, the droplets formed and left salt granules after drying. Through SEM, we confirm that 100 nm and even μm-scale particles are formed due to the high specific loading on the accumulated region (Figure S19). Either a substrate pretreatment, such as UV-ozone cleaning, or the adaptation of solvents that can infiltrate carbon can solve this issue, thereby achieving improved particle distribution on the substrate (Figure 5a). From the perspective of precursor library exploration, we suggest eliminating the use of aqueous solvents in FJH synthesis. Although the solvent does not participate in the decomposition reaction, nonaqueous solvents allow for a wide range of moisture-sensitive or water-insoluble organometallic compounds to be used as FJH precursor candidates.

Following these observations, we selected three different metal compounds with anions of chloride (Cl^–^), acetate (CH_3_COO^–^), and acetylacetonate (CH_3_COCHCOCH_3_^–^) as precursors for investigation. Transition metal chlorides exhibit a propensity for sublimation during FJH, as depicted in Figure 4a and Table S2. On the other hand, acetates demonstrate a superior capability to maintain the elemental ratio of the recipe, as shown in Figure 4c. Acetylacetonate precursors tend to lead to carbon deposits on the metals due to the increased carbon content of the precursor anion. In fact, we found that Pd–Cu–P nanoparticles prepared from copper­(II) acetylacetonate are fully covered by a thin layer of amorphous carbon (Figure S20).^49^ Electrochemical oxidation or UV–O_3_ etching increases the specific electrochemical surface area (Figure S21), suggesting that carbon encapsulation confined the surface area of the exposed catalysts and hinders the activity toward multiple reactions. Though carbon residue is not ideal for water electrochemistry, this hydrophobic carbon-based layer may be of interest to surface chemists for other studies.

Moreover, we discovered that the primary factor determining the average particle diameter is not the thermal profile but rather the combination of the alloying elements. To simplify the comparison and eliminate the complexity arising from the melting point variations caused by alloy composition, pure Pd was selected instead of a phosphide alloy to assess the impact of flashing time. During a 100 ms heating process, the attained *T*_max_ was only 517 K, which was insufficient for precursor decomposition. Consequently, no particles were formed, as depicted in Supplemental Figure S22a. When increasing the flashing time to 200 ms, the *T*_max_ reaches 1000 K where Pd reduction occurs,^50^ yielding particles with a 34.51 nm ± 12.77 nm mean diameter (Figure S22b). By further extending the flashing time to 2 s (Figure S22c–f), we demonstrate a mean diameter ca. 35 nm (Figure 5d,h).

The addition of multiple elements (P and Ni) can further reduce the particle size. When an equimolar amount of P precursor is added to the Pd precursor, the mean particle diameter reduces to 20.1 nm ± 2.4 nm (Figure 5e,i). The introduction of Cu further reduces the mean diameter to 11.2 nm ± 2.6 nm (Figure 5f,j). Finally, alloying Ni with Pd–P leads to the formation of 2 nm nanoclusters (Figure 5g,k). The incorporation of multiple elements could benefit the particle dispersion in several ways. First, the melting point of the alloy system is decreased, leading to a longer lifetime of melt droplets instead of solid particles. This enables Brownian motion of the melt droplets during the FJH process, as discussed by Klement et al.^51^ Second, following the melting-motion-fusion step (Figure 4b), the reduction in the droplet surface tension^52^ allows distribution of smaller residual nanoclusters. Third, enhanced mixing entropy is advantageous for stabilizing the liquid and lowers the critical cooling rate for glass formation.

### 2.4 Demonstrative Application

While the primary focus of this paper is on the synthesis and characterization of the FJH parameter space, we present a concise demonstration of the practical application potential of the as-synthesized MG nanoparticle composites. Specifically, we highlight their high efficiency in two pivotal processes: the oxygen evolution (OER) and methanol oxidation (MOR) reactions. These processes hold significant importance in the realms of water splitting and methanol fuel cells,^2^ respectively, and serve to showcase the broader applicability of our work. Literature has shown that MGs exhibit higher catalytic performance compared to their crystalline counterparts. With the successful synthesis of MG nanoparticles, our next goal is to test and optimize their catalytic properties such that we can prove the applicability of FJH as a platform for metallic glass catalyst development. Here we demonstrate the potential with our preliminary results from the newly synthesized MG nanoparticles. More detailed work focusing specifically on catalytic properties and material processing will be presented elsewhere.

For the OER tests, a free-standing MG loaded carbon strip was utilized as the working electrode, along with an Hg/HgO reference electrode and a Pt mesh counter electrode. We present the linear sweeping voltammogram (LSV) curves of representative crystalline and MG samples (Figures 6a and S23). The Pd–Ni alloys exhibit a lower onset potential (1.62 V vs RHE) compared to pure Pd (1.68 V vs RHE), indicating improved catalytic activity resulting from the addition of nickel. On the other hand, Cu alloying was found to be detrimental, leading to a decrease in current density. Among all the samples studied, the MG Pd_1_Ni_4_P alloy (Pd_22_Ni_70_P_8_ measured by EDX) achieved the highest current density of 80 mA cm^–2^ at 1.74 V vs RHE.

**Figure 6:**
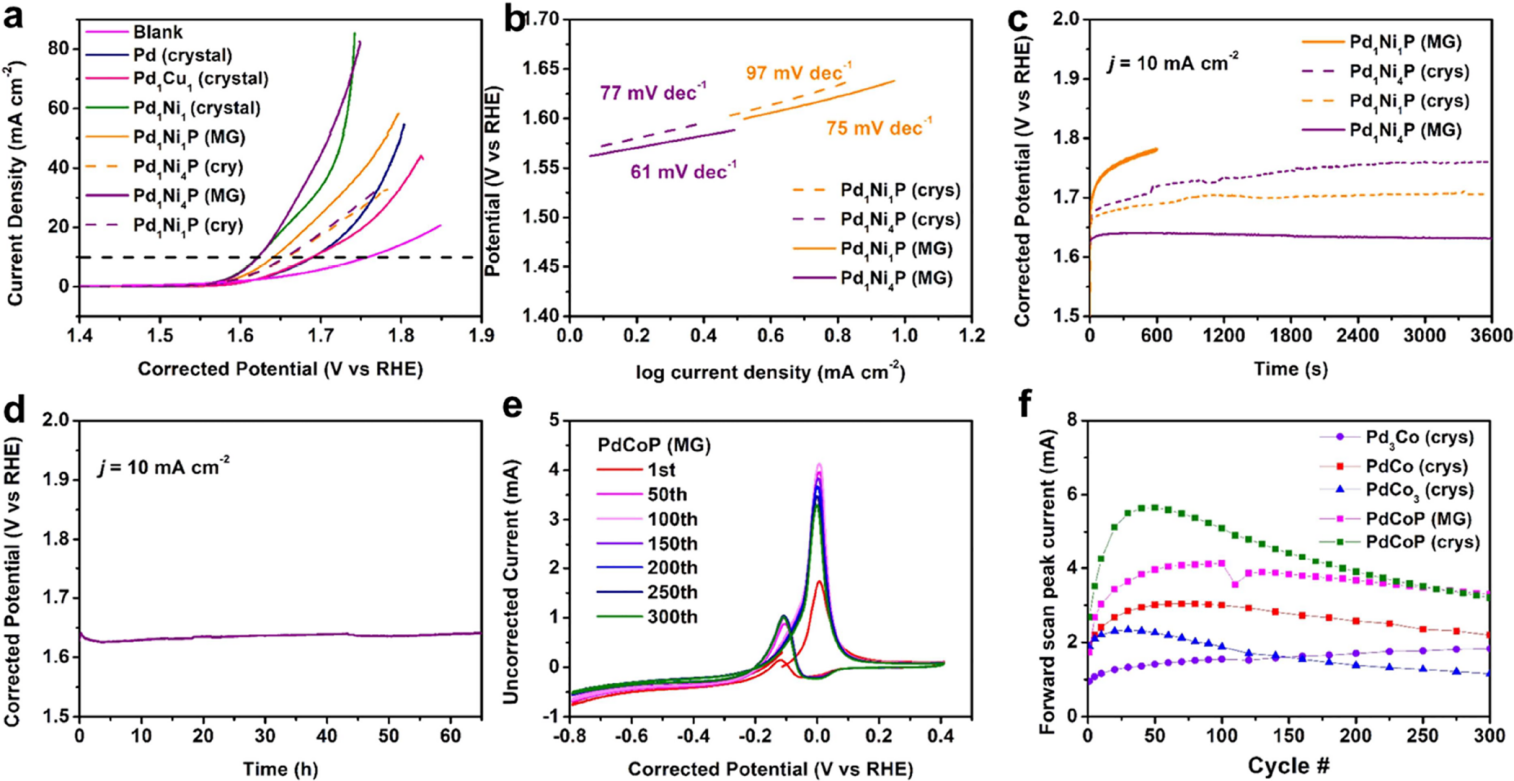
Electrocatalytic performance of amorphous metal phosphide nanoparticles. (a) OER linear sweeping voltammogram scans. “Crystal” and “MG” refers to the crystalline and glassy phase respectively, which was characterized by XRD. (b) OER Tafel slope and (c) chronopotentiometry of representative FJH synthesized alloys. (d) Long-term OER stability test of glassy Pd_1_Ni_4_P. (e) MOR catalysis of MG PdCoP and (f) stability test of various MG samples and crystallized counterparts.

To understand the role of the amorphous structure while maintaining the overall particle composition, the OER activity of crystallized particles was also investigated. The Pd–Ni–P alloys were crystallized by annealing at 1073 K for 2 h and cooled at a rate of 0.1 K s^–1^, as confirmed by the increasing XRD diffraction peak intensity (Figure S24). We show that the recrystallized Pd–Ni–P nanoparticles exhibit a decline in overall activity, as evidenced by the increasing overpotential (1.65 V) and decreasing current density (Figure 6a). The Tafel slopes, calculated from LSV scans, increase for all crystalline samples, indicating slower kinetics for electron transfer (Figure 6b).^53^

Furthermore, chronopotentiometry results validate the exceptional stability of amorphous Pd_1_Ni_4_P, as it maintained a stable potential of 1.6 V vs RHE for the duration of the experiment, while the potentials of the other alloys continued to increase (Figure S25). The potentials of the crystallized samples exhibit a progressive increase during stability testing (Figure 6c). No catalyst deactivation of the amorphous Pd–Ni–P was observed throughout the 60-h long-term stability test (Figure 6d). XRD (Figure S26) and SEM (Figure S27) analyses confirm the preservation of the amorphous structure in the Pd_1_Ni_4_P metallic glass after the stability test. Both voltammograms and chronopotentiometry results provide direct evidence of the catalytic advantages offered by the glassy materials over their crystalline counterparts, which aligns with existing literature.^4^ FJH presents itself as a method for creating amorphous catalysts that can leverage the benefits of a glassy structure while maintaining nanoparticle size distribution.

To test the versatility of the MG alloys created using FJH, we also ran the MOR to compare crystalline versus amorphous catalyst behavior. The catalytic performance for the methanol oxidation reaction was assessed using cyclic voltammetry (CV) in 1 M KOH + 1 M methanol aqueous solution (Figures 6e and S28). In a typical MOR CV scan, a Pd-based catalyst typically exhibits two oxidative peaks. The forward scan peak arises from the oxidation of methanol, while the reverse scan peak is attributed to the removal of intermediates that were not fully oxidized in the forward scan.^6,7^ Among all the synthesized catalysts, amorphous PdCoP exhibits the highest forward scan peak current, indicating a faster reaction rate and superior activity. The forward scan peak current of PdCoP increases from 1.5 mA during the initial 50 cycles, indicating catalyst activation, and remains at approximately 3.9 mA for the subsequent 250 cycles. No XRD peaks were observed after the electrocatalytic testing, suggesting the absence of crystallization during catalysis runs (Figure S29). The catalytic stability was verified through accelerated durability testing (Figure 6f). Based on the forward scan peak current, it is evident that the addition of phosphorus to Pd–Co alloys significantly enhances both the activity and stability. Crystallized PdCoP was also prepared through thermal annealing in a tube furnace. While the crystalline counterpart exhibited a higher peak current during the initial 100 cycles, it experienced rapid activity decline that was not experienced as drastically in amorphous samples.

## 3 Conclusions

In summary, we present flash Joule heating as an easy, versatile method for synthesizing metallic glass nanoparticles with enhanced control over particle size, composition, and morphology. The ability to quickly reach high temperatures enables efficient precursor decomposition, while the rapid cooling process leads to the formation of metastable amorphous nanoparticles. Our study demonstrates the effectiveness of this method in synthesizing various alloy systems (Pd–P, Pd–Ni–P, Pd–Cu–P, and Pd–Co–P), resulting in products with completely amorphous structures. Precise control over composition and desired particle size distribution can be easily achieved by manipulating variables such as the precursor recipe and heating conditions. Furthermore, we provide evidence of the superior catalytic activity of these as-synthesized amorphous materials compared to their crystalline counterparts in both the oxygen evolution and methanol oxidation reactions. The results presented in this study highlight the application of flash Joule heating as a selective area heating strategy, providing informative guidance for future amorphous materials design and synthesis.

## 4 Materials and Methods

### 4.1 Materials Preparation and Synthesis

Toray carbon fiber paper 060 20 × 20 cm (SKU:591037, Lot#2651-2) was purchased from the Fuel Cell Store. A carbon paper strip with 60 mm in length and 2 mm in width was cut by razor blades and suspended above a piece of microscopic slide (Fisher, Catalog #12-550-016) by connecting it to two copper electrodes with conductive copper tape.

For the standard synthesis, palladium dichloride (PdCl_2_, Sigma-Aldrich), nickel dichloride (NiCl_2_, Sigma-Aldrich), cupric acetate (Cu­(CH_3_COO)_2_, Sigma-Aldrich) were used as metal precursors. Solutions of 0.1 mol/L of each metal precursor was prepared by dissolving in DI water. The 0.05 mol/L PPh_3_ in ethanol solution was used for P doping. For the sample loading, a precursor metal aqueous solution was first mixed based on the desired ratio by stirring. Then, 20 μL was directly dropped onto the suspended carbon film (20 mm × 2 mm), reaching a loading of 50 μL/cm^2^. After drying at room temperature, 20 μL 0.05 mol/L PPh_3_ in ethanol solution was loaded onto the suspended carbon film as well. These precursor-loaded carbon films were used directly for the FJH Synthesis. To synthesize the samples with varying Pd/P ratios (Supplementary Figure 7), the volume of PdCl_2_ solution was kept constant and the volume of PPh_3_ was adjusted accordingly. For the precursor comparison made in Supplementary Figure 17, Cu­(CH_3_COO)_2_ was replaced by CuCl_2_ and copper acetylacetonate Cu­(acac)_2_.

The FJH process was achieved by electrically triggered Joule heating of the precursor loaded carbon fiber strip in an argon-filled glovebox. A thermal shock was applied to the carbon strip by an Arduino-controlled relay. The shock duration time was controlled by a mechanical relay and the current was controlled by the power supply voltage. A 15 V 2000 ms electrical pulse was chosen as the ideal thermal shock duration and used to synthesize metallic glass nanoparticles in this work (unless noted otherwise).

Crystallization of metallic glasses was conducted in a tube furnace. The sample strips were transferred into a ceramic tube with Argon purging for 2 h before heating. The sample was heated at 1073 K for 2 h with a ramping rate of 5 K/min. The sample was collected after cooling to room temperature.

### 4.2 Material Characterization

X-ray diffraction (XRD) was performed on a Bruker AXS D8 discover GADDS micro diffractometer. The carbon fiber strip containing loaded materials was directly placed on the sample holder for signal collection. Signals were converted to patterns using Pilot software. Scanning electron microscopy (SEM) was performed on a MERLIN field emission scanning electron microscope. A 2 mm × 2 mm piece was cut from the reactor and attached to the sample plate using carbon tape without any gold sputtering. SEM energy dispersive X-ray analysis (EDX) was collected on the MERLIN SEM-EDX mode and analyzed using Aztec software. Thermographic analysis was conducted with a FLIR thermographic camera. The camera was factory calibrated and the working distance was set to 20 cm.

X-ray photoelectron spectroscopy (XPS) utilized a Physical Electronics Versaprobe II XPS. A 2 mm × 4 mm piece was cut from the reactor and attached to the sample plate using double side tape. Electron neutralization was enabled and presputtering was disabled. The XPS data was analyzed using Multipak software. Transmission electron microscopy (TEM) and selected area electron diffraction (SAED) were performed on a 200 kV Titan Themis TEM with Cs image corrector. The selected area was 40 nm. Scanning transmission electron microscopy (STEM) EDX was conducted on the STEM mode using Espirit software.

### 4.3 Electrochemical Testing

The OER activity was tested with a three-electrode set up. The electrolyte used was N_2_-saturated 1 M KOH aqueous solution. The metallic glass loaded carbon strip was used as a free-standing working electrode, a mercury/mercury oxide (MMO) electrode as reference, and a Pt mesh as the counter electrode. The reference electrode was calibrated with a reversible hydrogen electrode. Similarly, the MOR activity was tested using O_2_-saturated 1 M KOH + 1 M methanol aqueous solution electrolyte. The scan rate for MOR CV was 5 mV s^–1^. Stability was characterized by an accelerated durability test (ADT) with 300 cycles CV scanning.

$$E^{⊖}(\mathrm{MMO})=0.1095\mathrm{VvsRHE}$$

Electrochemical impedance spectroscopy (EIS) was used to determine the uncompensated solution resistance, typically in the range from 10 to 30 Ω in all cases. The ohmic-drop was compensated for in the voltammograms and chronopotentiometry curves of all samples. Voltammograms were measured by scanning from open circuit to 1.8 V vs MMO at the rate of 5 mV s^–1^. Chronopotentiometry was measured by setting current as 10 mA cm^–2^.

### 4.4 Pd–Ni TTT Diagram in Molecular Dynamics Simulation

The vitrification and crystallization behavior of Pd–Ni alloys was studied via MD simulation with an aim to obtain a time–temperature-transformation (TTT) diagram for the metallic glass formation in several metallic systems related to FJH. From the critical nose time obtained from the MD simulations, we can map out the range of the critical cooling rates in the FJH process.

To study how the composition and alloy concentration affects glass formation, we used pure Pd and Ni, and alloys Ni_5_Pd_95_, Ni_10_Pd_90_, Ni_15_Pd_85_, Ni_20_Pd_80_, and Ni_25_Pd_75_. As crystallization takes longer time with increasing nickel content in the alloy system, we focused on the five limited Pd–Ni binary systems to understand the general trend.

To simulate cooling and the related atomic-level properties, we use the classical MD simulation implemented via the large-scale atomic/molecular massively parallel simulator (LAMMPS).^54^ Pd, Ni, and Pd–Ni atoms interact with the embedded atom method (EAM) interatomic interactions.^55^ This interaction has been used in various occasions of cooling and isothermal simulation of glass forming processes.^56^ Several systems of different size were tested that contain a different number of atoms ranging from 62,500, 108,000 to 500,000 atoms with periodic boundary conditions in three directions. For both pure metals and alloys, all data obtained from the largest sample with 500,000 atoms is shown in this paper. The isothermal–isobaric (NPT) ensemble MD was applied to achieve the target temperature during continuous cooling and isothermal process controlling the pressure of the system at zero pressure in all simulations. The temperature is controlled by the Nosé-Hoover algorithm.^57^ The Newton equation of motion was solved numerically with a time step of 1 fs per step. After equilibrating the liquid at 2500 K for 1 ns, the systems are continuously quenched with cooling rate of 5 × 10^13^ K/s to a series of designated temperatures for 1–10 ns to study isothermal crystallization.

The TTT diagrams were constructed from the isothermal crystallizations, which are shown in Supplementary Figure 13. They are obtained by holding the liquids at various temperatures and detecting the time when a crystallization event occurs. Supplementary Figure 13 shows the incubation time for crystal nucleation. For pure metals (Pd and Ni), the crystallization takes place within a fraction of nanosecond over a wide temperature range down to 500 K. The crystallization of Ni is faster than that of Pd. The nose of the TTT diagram is about 850 K for Ni and about 900 K for Pd. For the Pd–Ni binary systems, the simulation results are well reproduced from 600 to 1100 K. The nose of the TTT diagram remains at about 900 K for all samples with varying Ni concentrations. However, the crystallization occurs with longer incubation time with the increasing Ni concentration over a narrow temperature range as shown in the shift of the TTT diagrams to the right. It becomes more difficult to detect the nucleation at high temperatures owing to the long incubation time. Similarly, when the concentration of Ni is larger than 15%, it becomes difficult to detect the crystallization below 700 K due to significant kinetic constraints on growth in the supercooled liquids.

The TTT diagram allows us to calculate the approximate critical cooling rate using the minimum incubation time, i.e., the nose time, and the corresponding temperature in Supplementary Figure 13. The change of nose time versus Ni concentration is shown in Supplementary Figure 14. The crystallization time increases with Ni concentration *x*; we fit the time dependence of Ni with an exponential function *t* = *t*_0_ + *A*·exp (*B*·*x*). The fitted parameters *t*_0_, *A* and *B* are 98.70, 0.48, and 0.38 respectively. The unit of fitting time is picosecond. From the fitted formula, we can estimate the critical crystallization time at Ni concentrations larger than 25% which is too long to simulate with modern computers. For example, *t* is about 6.21 ns at *x* = 25% and 40.59 ns at *x* = 30% but approaches 1.7 μs when Ni is larger than 40%. The estimated critical time and cooling rate for the glass formation are not far apart as those achievable in FJH.

## Supplementary Material



## Notes

Derivation of the FJH thermal profile; FJH circuit diagram for controlling voltage pulse; theoretical thermal profile during FJH calculated from Newton’s Law of cooling and Joule’s Law; SEM-EDX spectrum and elemental maps of the Pd_3_P alloy; XRD spectra of Pd–P alloys with varying Pd/P ratios; SEM images of PPh_3_ on the FJH substrate; SEM images and EDX spectra of nanoparticles of varying Pd/P ratio; STEM-EDX map and XPS spectra of Pd–Ni–P alloy; XRD spectra of Pd–Ni–P alloys with varying P ratios; XRD spectra and SEM images of PdNiP alloys with 10 and 15 μmol/cm^2^ loading; molecular dynamics simulated TTT diagrams for Pd, Ni, and Pd–Ni alloys; nose time and critical cooling rate for Pd–Ni alloys; SEM-EDX spectra of Ni–P alloys; XPS spectra of Pd_70_Cu_9_P_20_ alloy; XRD spectra of Pd–Cu–P alloys with varying Pd/Cu ratio; image of NiCl_2_ precursor droplets on the hydrophobic carbon fiber substrate; SEM images of the nonuniform particle distribution caused by surface hydrophobicity; SEM images of Pd–Cu–P nanoparticles derived from copper­(II) acetylacetonate; cyclic voltammetry of Pd–Cu–P nanoparticles before and after OER testing; SEM images and corresponding nanoparticle size distributions of Pd nanoparticles based on flash time; LSV of Pd3P and Pd–Cu–P alloys; XRD spectra of amorphous and recrystallized Pd–Ni–P and Pd–Cu–P alloys; OER stability test results of various Pd–Ni–P samples; XRD spectra and SEM images before and after OER stability testing of PdNi_4_P alloy; MOR performance of Pd–Ni–P and Pd–Cu–P alloys; XRD spectra of recrystallized and amorphous Pd–Co–P alloys; ramping and cooling rate calculations; numerical EDX elemental analysis of various Pd–Ni–P and Pd–Cu–P alloys; and digital and thermographic videos of the flash Joule heating process (PDF)
