# Supplementary material for: Metallic Glass Nanoparticles Synthesized via Flash Joule Heating
Source: ACS Nano. 2025 May 15;19(21):19806–17. doi: 10.1021/acsnano.5c02173 (PMC12139038; doi:10.1021/acsnano.5c02173)
Supplement: Supplementary file 1 [file nn5c02173_si_001.pdf]

Supplementary Information

Metallic Glass Nanoparticles Synthesized via Flash Joule Heating

Hang Wang[1], Nathan Makowski,[1] Yuanyuan Ma[1], Xue Fan[2], Stephen A. Maclean[1], Jason Lipton[1], Juan Meng[1], Jason A. Röhr[1], Mo Li[3], and André D. Taylor[1,*]

[1]Department of Chemical and Biomolecular Engineering, Tandon School of Engineering, New York University, New York, NY 11201, USA

[2] College of Materials, Shanghai Dianji University, Shanghai 201306, China

[3]School of Material Science and Engineering, Georgia Institute of Technology, Atlanta, GA, 30332, USA

*Corresponding Author: andre.taylor@nyu.edu

This PDF file includes:

Supplementary Text
Figures. S1 to S29
Tables S1 to S2
Captions for Movies S1 to S2

Other Supplementary Materials for this manuscript include the following:

Movies S1 to S2

Supplementary Text

Derivation of substrate thermal profile under ideal conditions

A thermodynamic calculation is carried out to analyze the ramping rate and maximum temperature. Annotations are listed below:

Independent Variables	Power Supply Voltage	U
	Flashing Time	t
	Substrate Length	L
	Substrate Width	W
Known constants	Substrate thickness	H
	Substrate Resistivity	P
	Environmental temperature	T_{enviro}
	Substrate Density	D
	Heat Capacity	C_p
	Heat transfer coefficient	H
Derivable variables	Volume	V = L * W * H
	Current	I = U / R
	Area	S = L*W
	Mass	m = D * V
	Resistance	$R = \frac{\rho * L}{W * H}$
	Initial temperature before cooling (from heating process)	$T_{initial}$

With no evaporation or degradation and assuming that ρ, D, h, C_p are constants, the heat dissipation follows the Newton's cooling law. For heating, the heat flow, heating rate as well as the temperature variation are described by the following relations,

$$\frac{dQ}{dt} = m * C_p * \frac{dT}{dt} = I^2 * R - h * S * (T - T_0)\frac{dT}{dt} = \left(\frac{I^2 * R}{m * C_p}\right) - \left(\frac{h * S}{m * C_p}\right) * (T - T_0)$$

$$T(t) = T_{enviro} + \frac{H}{h*\rho} * \left(\frac{U}{L}\right)^2 * \left[1 - exp\left(-\frac{h}{D*H*C_P} * t\right)\right].$$

And for cooling,

$$\frac{dQ}{dt} = m * C_p * \frac{dT}{dt} = -h * S * (T - T_0)$$

$$T(t) = T_{enviro} + (T_{initial} - T_{enviro}) * exp\ (-\frac{h}{D * H * C_P} * t)$$

The Thermal profile, i.e., the temperature versus time, obtained for the strip in our FJH is shown in Fig. S2.

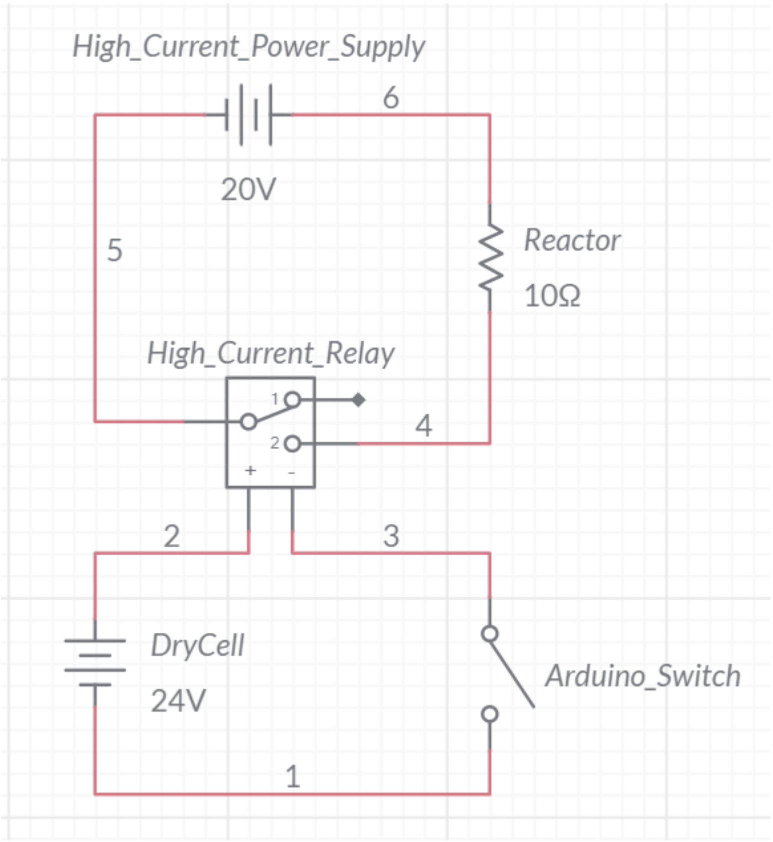

Figure S1. The sketch drawing of self-designed flash Joule heating system

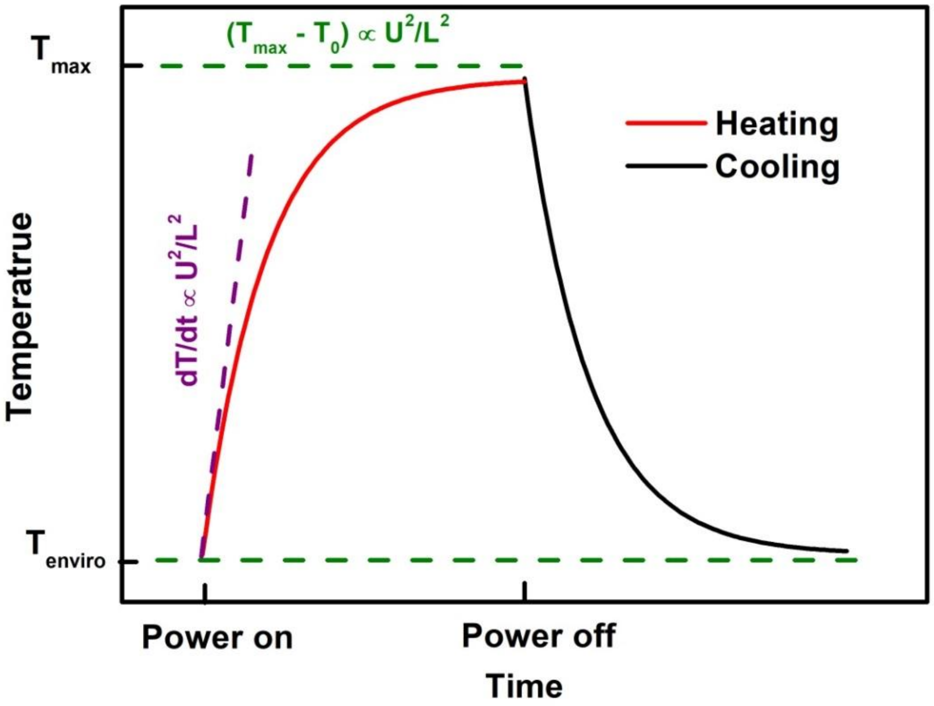

Figure S2. The theoretical thermal profile of flash Joule heating predicted from Newtonian Law of cooling and Joule's Law

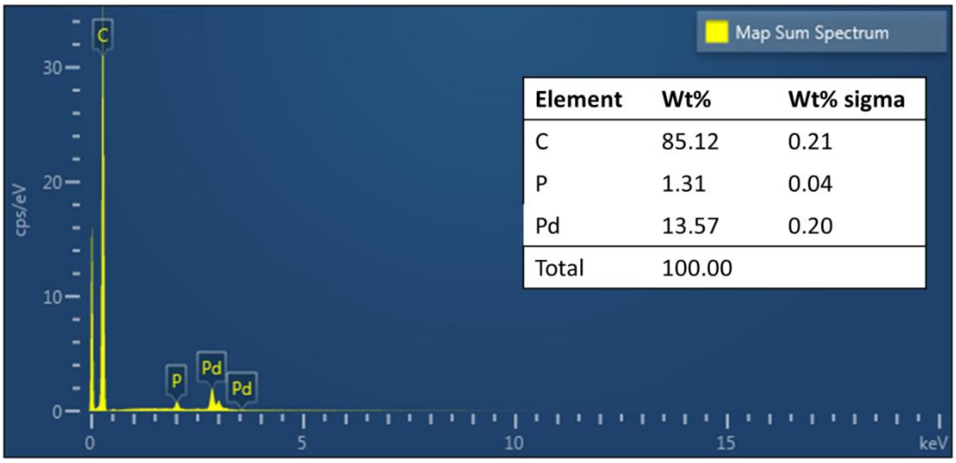

Element	Wt%	Wt% sigma
C	85.12	0.21
P	1.31	0.04
Pd	13.57	0.20
Total	100.00	

Figure S3. SEM-EDX spectrum of binary Pd_3P. Inset: elemental analysis.

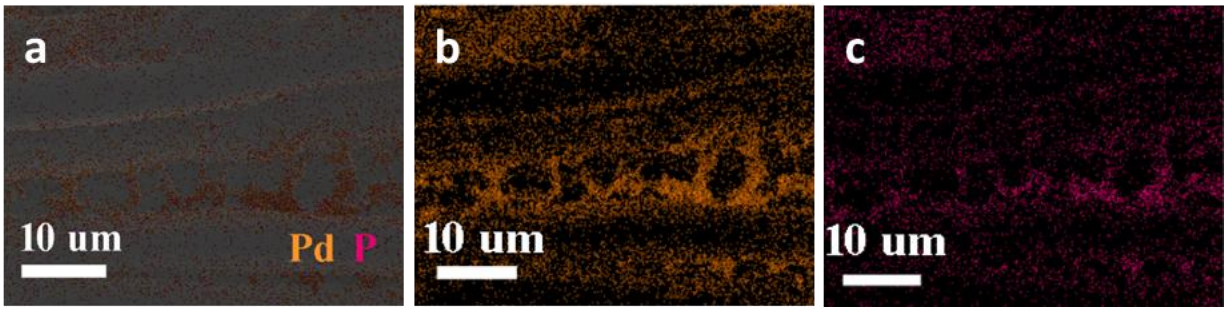

Figure S4. SEM-EDX mapping of Pd_3P: a) Mixing, b) Palladium, and c) Phosphorus.

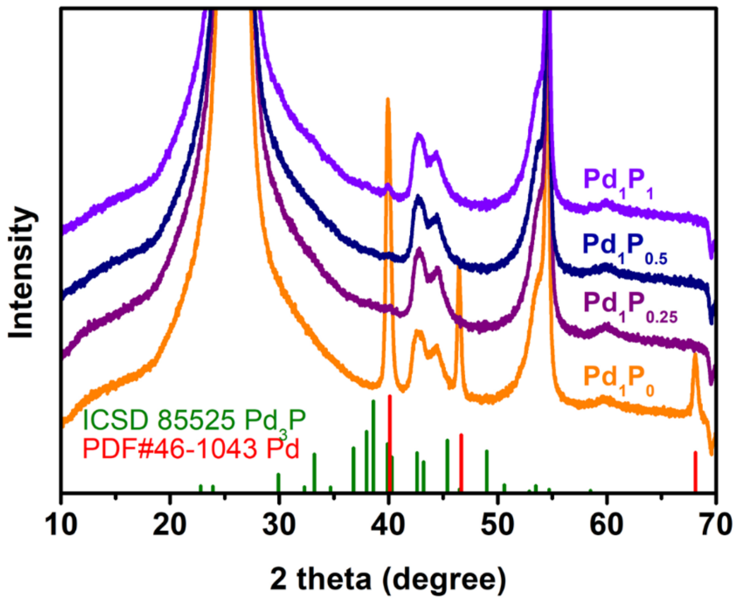

Figure S5. XRD patterns of Pd-P alloy with varying Pd/P ratios.

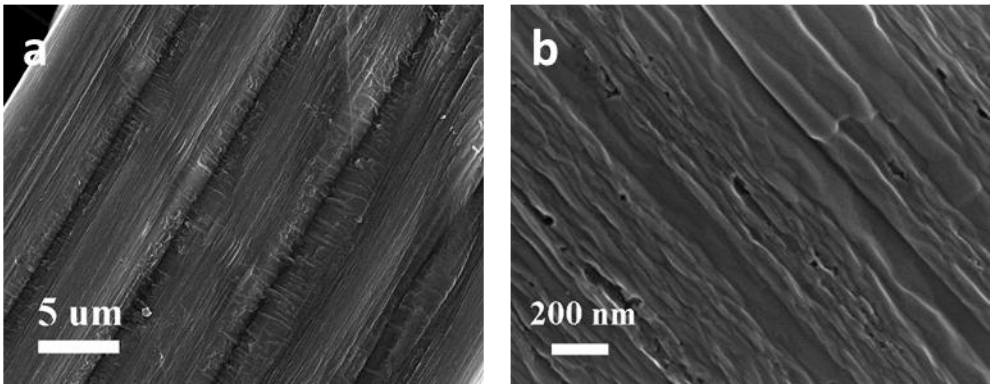

Figure S6. SEM image of FJH heated PPh_3 sample. a) SEM image at low magnification, b) SEM image at high magnification.

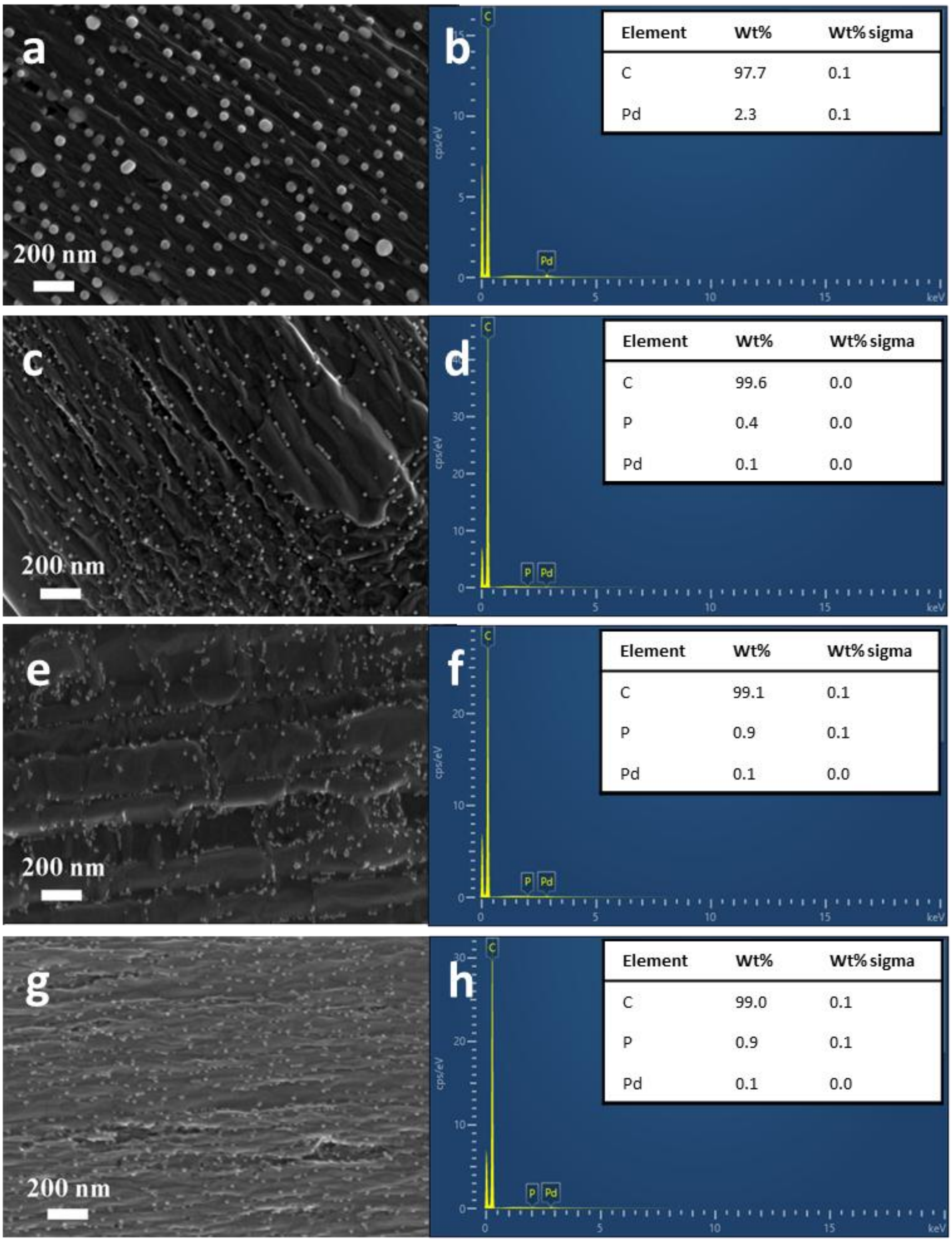

Figure S7. SEM images for varying Pd/P ratio. **a,** P/Pd=0, **c,** P/Pd=1/4; **e,** P/Pd=1/2; **g,** P/Pd=1/1. **b, d, f, h** SEM-EDX spectrum and elemental analysis of Pd-P alloys correspondingly. Other Pd-P compounds are formed with low P/Pd ratio.

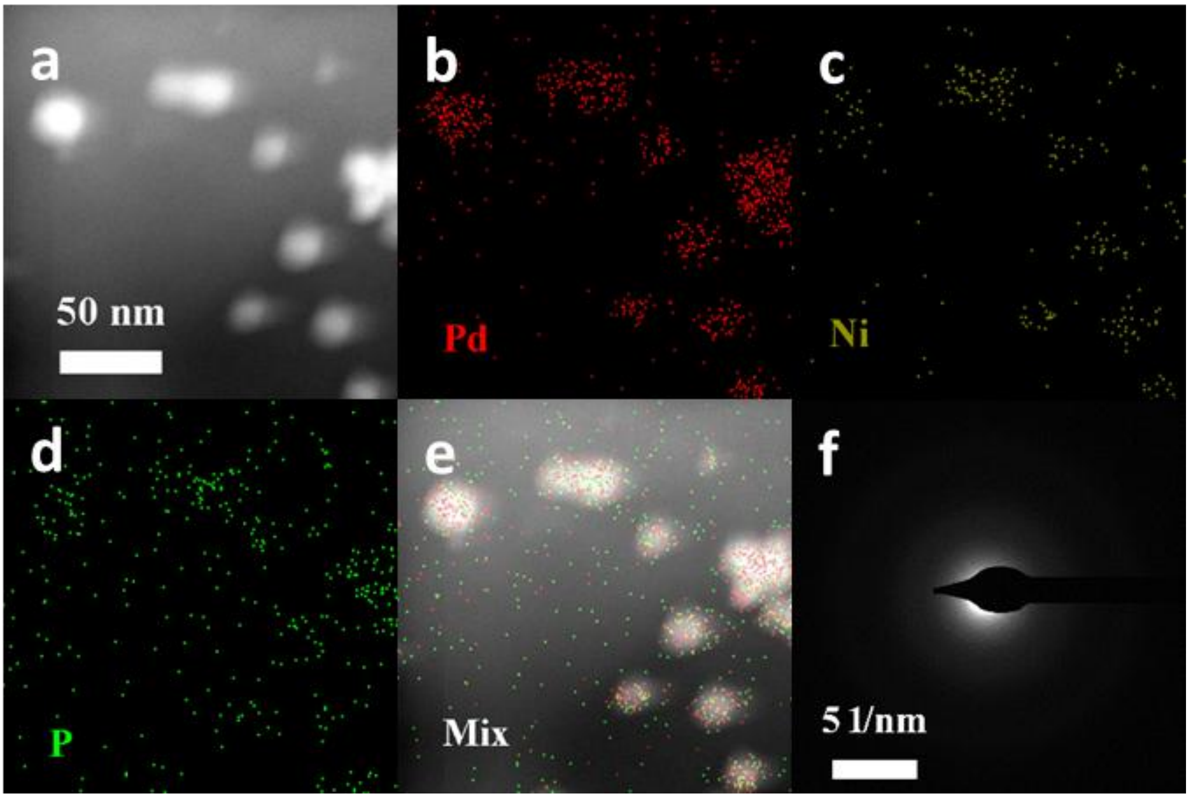

Figure S8. STEM-EDX mapping of $Pd_{58}Ni_{31}P_{11}$ alloy. **a,** HADDF images. **b,** Pd, **c,** Ni, **d,** P and **e,** overlapped mapping of amorphous $Pd_{58}Ni_{31}P_{11}$ nanoparticles. **f,** SAED pattern of a $Pd_{58}Ni_{31}P_{11}$ nanoparticle.

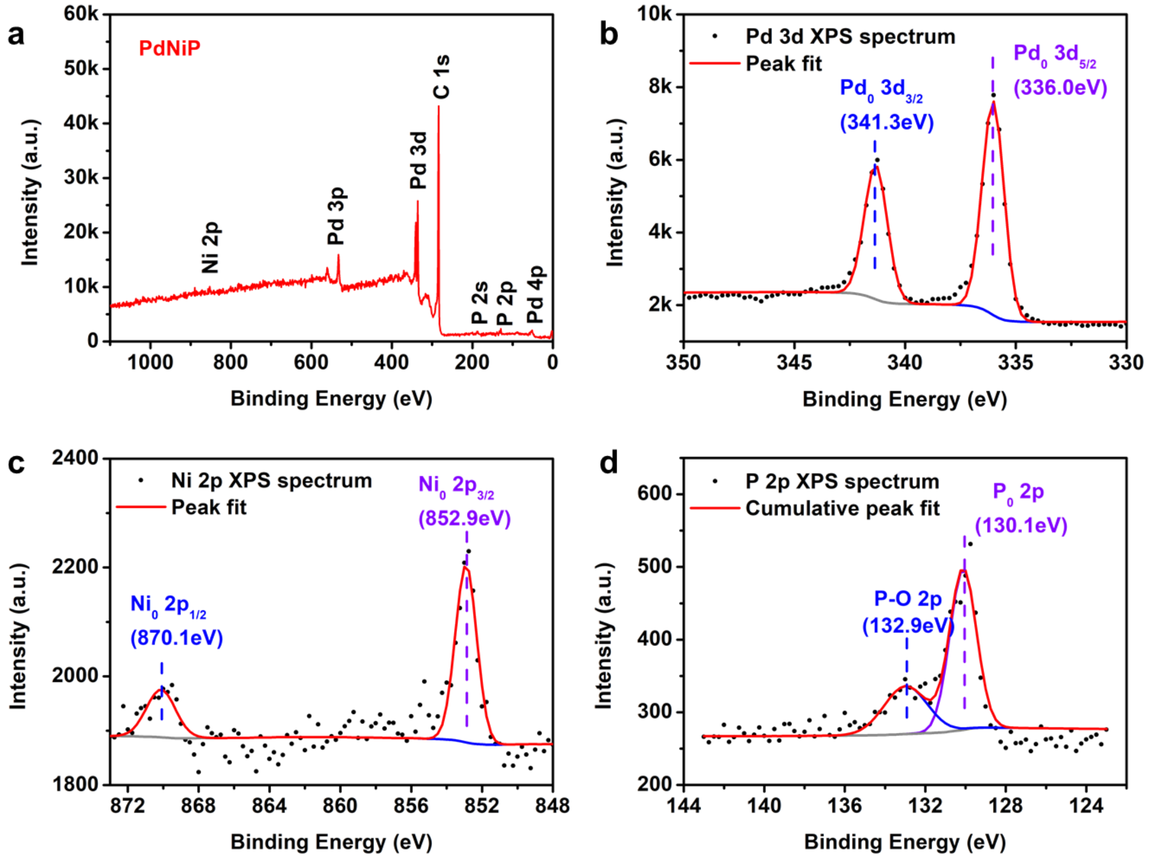

Figure S9. a, XPS spectrum of $Pd_{58}Ni_{31}P_{11}$ and high resolutions peaks for **b,** Pd 3d, **c,** Ni 2p and **d,** P 2p.

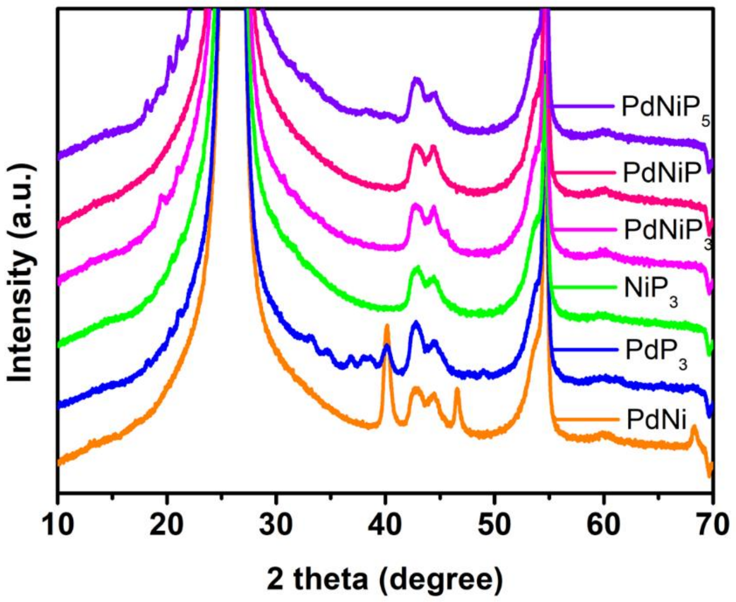

Figure S10. XRD patterns of Pd-Ni-P alloys with varying P ratios.

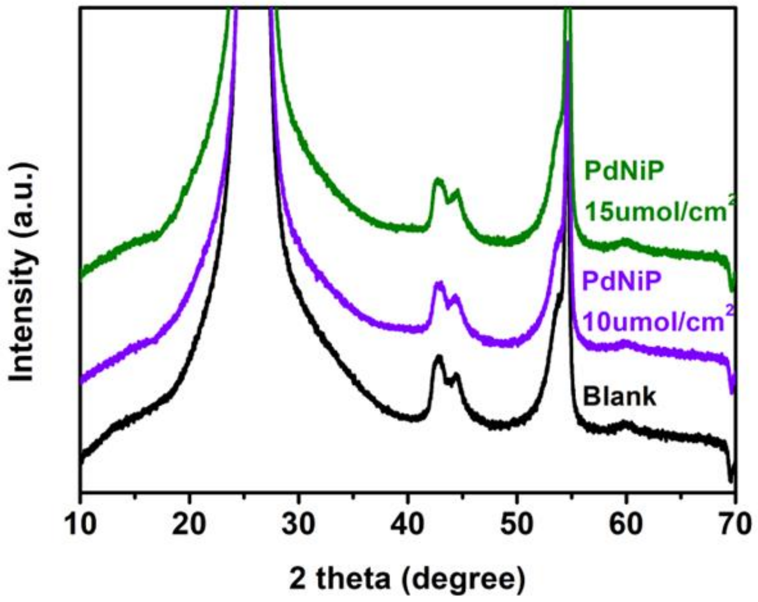

Figure S11. XRD patterns of PdNiP 10 μmol/cm^2 and PdNiP 15 μmol/cm^2.

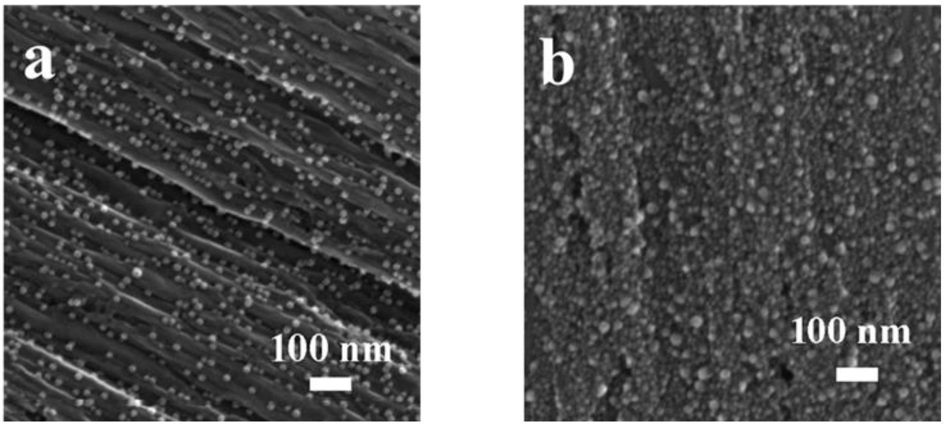

Figure S12. SEM images of **a,** PdNiP 10 μmol/cm^2 and **b,** PdNiP 15 μmol/cm^2.

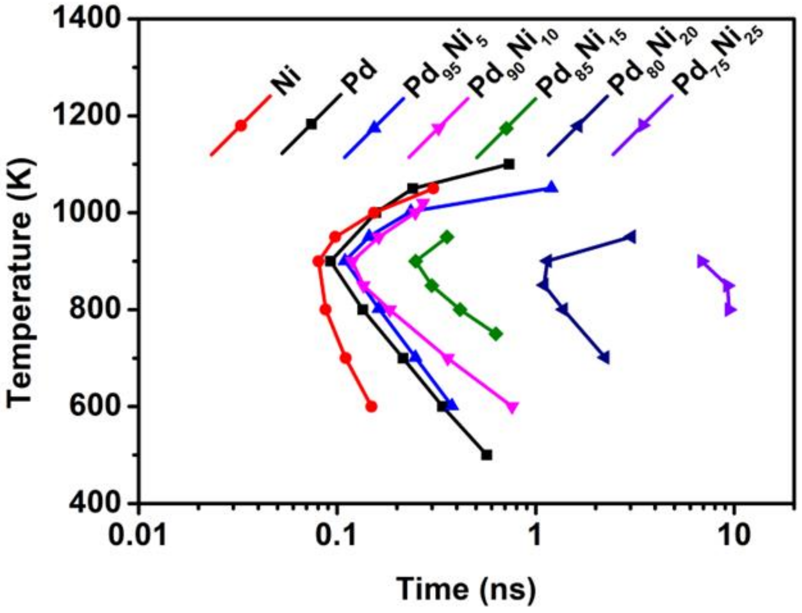

Figure S13. Molecular dynamics simulated TTT diagrams for Pd, Ni and PdNi. The lines are the guide for the eye.

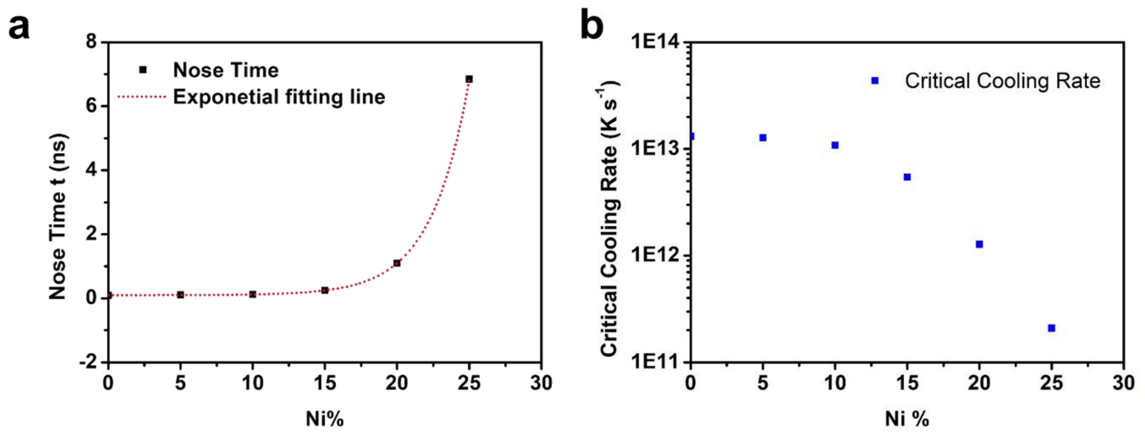

Figure S14. a, The nose time and **b,** critical cooling rate of Pd-Ni alloys versus Ni concentration. The data points (filled squares) are taken from Fig. S13.

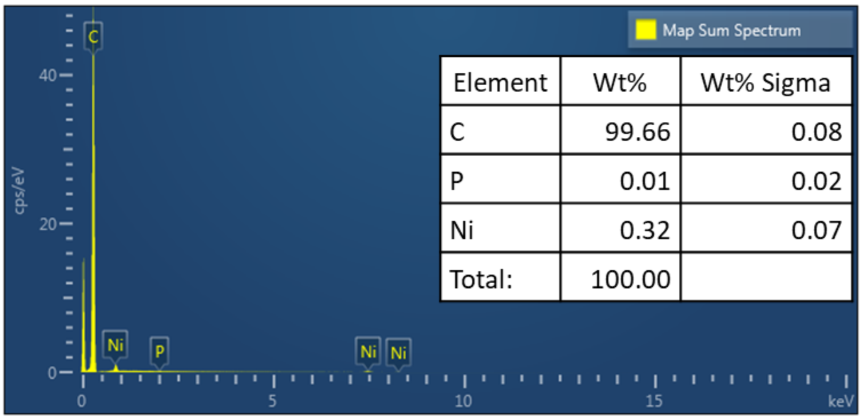

Figure S15. SEM-EDX spectrum of Ni-P alloys. Inset: elemental analysis.

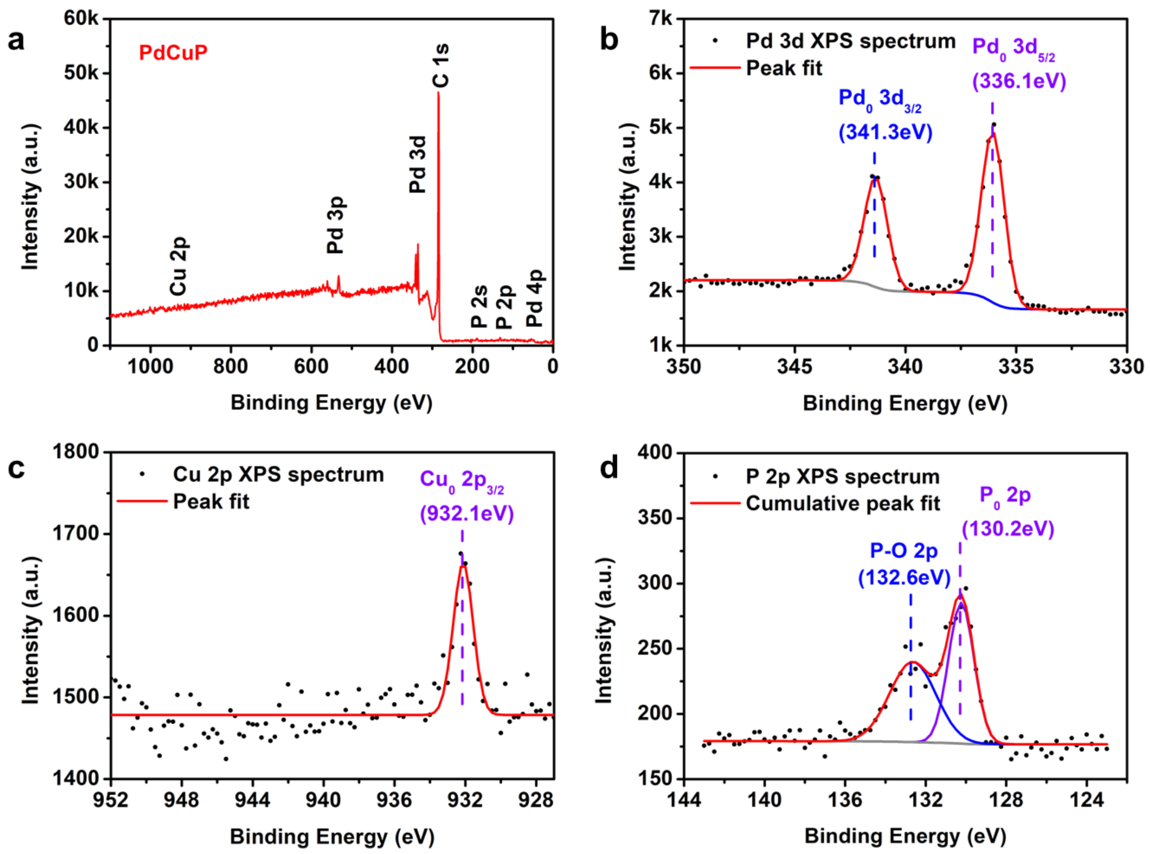

Figure S16. a, XPS spectrum of $Pd_{70}Cu_9P_{20}$ and high resolutions peaks for **b,** Pd 3d, **c,** Cu 2p and **d,** P 2p.

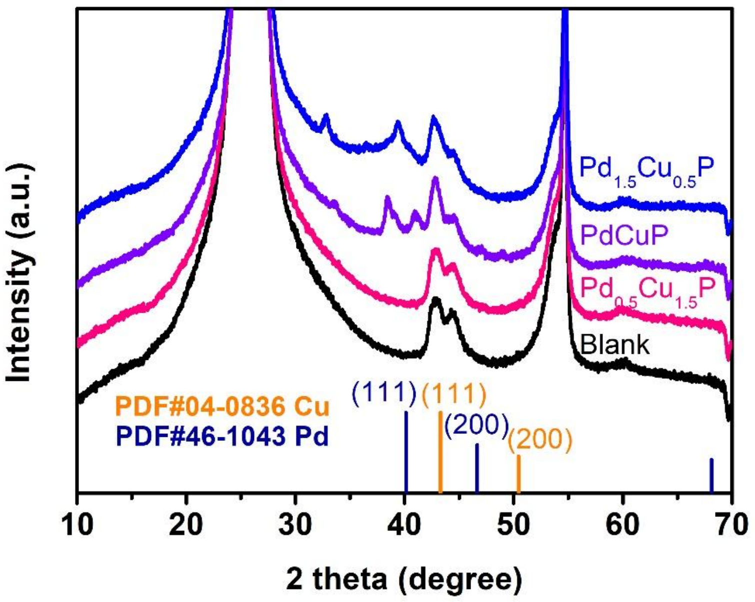

Figure S17. XRD patterns of Pd-Cu-P alloys with varying Pd/Cu ratio.

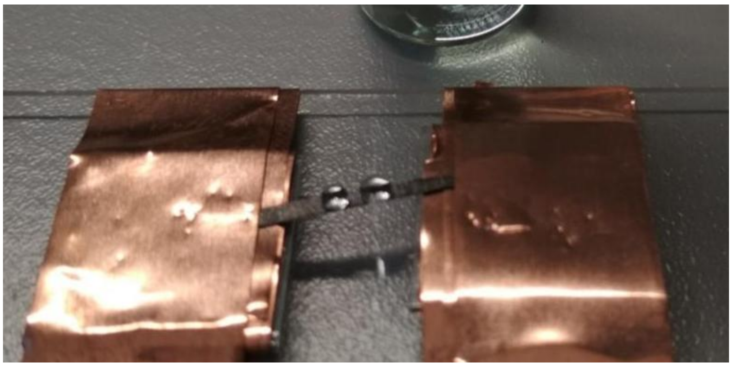

Figure S18. Formation of precursor solution droplets on hydrophobic substrate. The solution was 0.05M $NiCl_2$ in water and the substrate was hydrophobic carbon fiber paper.

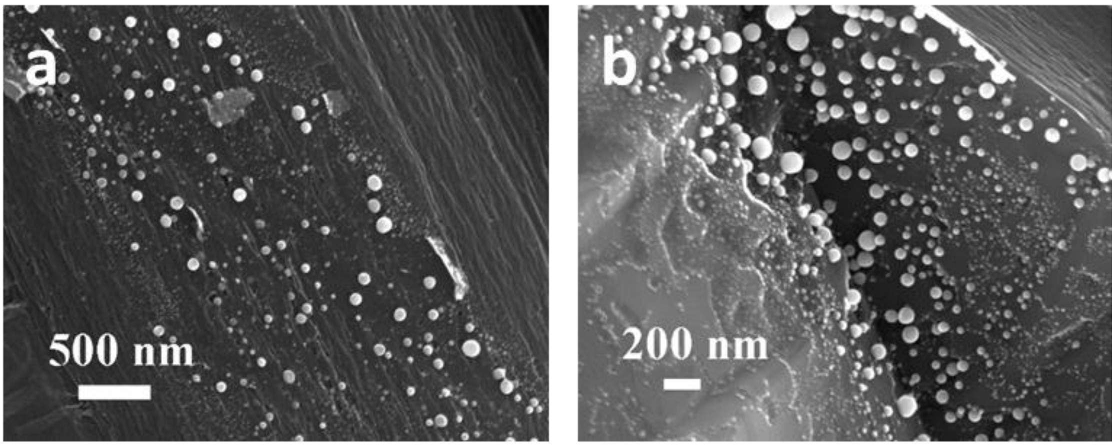

Figure S19. Non-uniform Pd-Ni particle distribution due to substrate surface hydrophobicity. **a,** SEM image at low magnification, **b,** SEM image at high magnification.

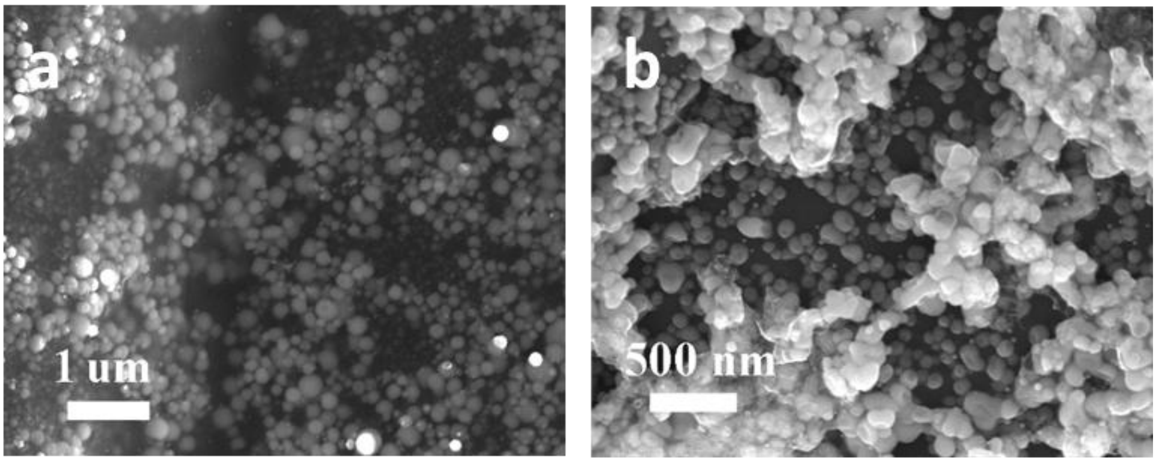

Figure S20. SEM images of carbon deposit on the PdCuP alloy nanoparticles when using copper (II) acetylacetonate as the precursor. **a,** SEM image at low magnification, **b,** SEM image at high magnification.

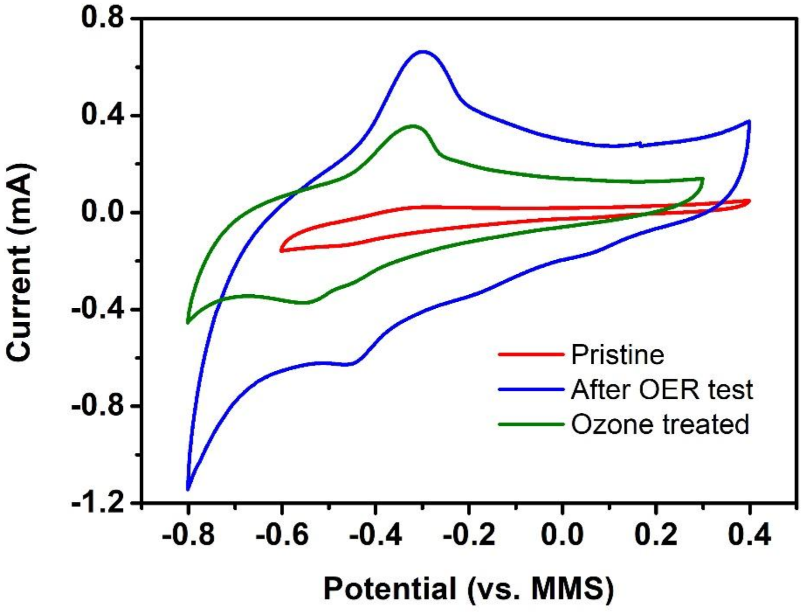

Figure S21. Cyclic voltammetry scans of PdCuP alloy nanoparticles synthesized from copper(II) acetylacetonate. The scan rate was 50 mV/s. The electrolyte was N_2-saturated 0.5M H_2SO_4. The reference electrode was mercury/mercury sulfate (Hg/$HgSO_4$, MMS). "Pristine" refers to the pristine PdCuP alloy nanoparticles synthesized from copper(II) acetylacetonate. "After OER tests" refers to the PdCuP alloy nanoparticles after 5 cyclic voltammetry scans from 0 V vs MMS to 1.0 V vs MMS. "UV-Ozone treated" refers the PdCuP alloy nanoparticles treated by 5 min UV-ozone treatment before testing.

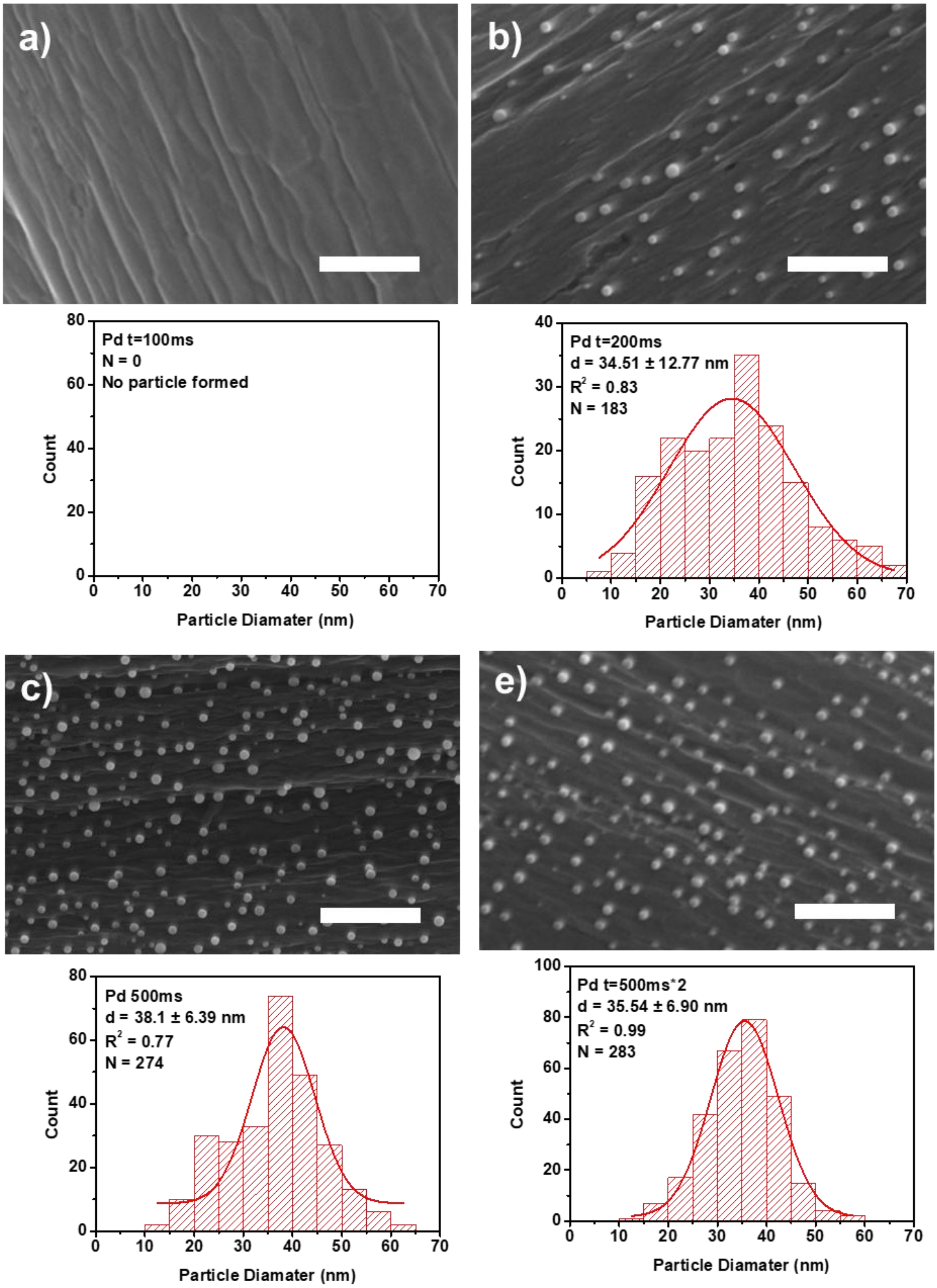
a)
Pd t=100ms
N = 0
No particle formed
Count
Particle Diamater (nm)
b)
Pd t=200ms
d = 34.51 ± 12.77 nm
R² = 0.83
N = 183
Count
Particle Diamater (nm)
c)
Pd 500ms
d = 38.1 ± 6.39 nm
R² = 0.77
N = 274
Count
Particle Diamater (nm)
e)
Pd t=500ms*2
d = 35.54 ± 6.90 nm
R² = 0.99
N = 283
Count
Particle Diamater (nm)

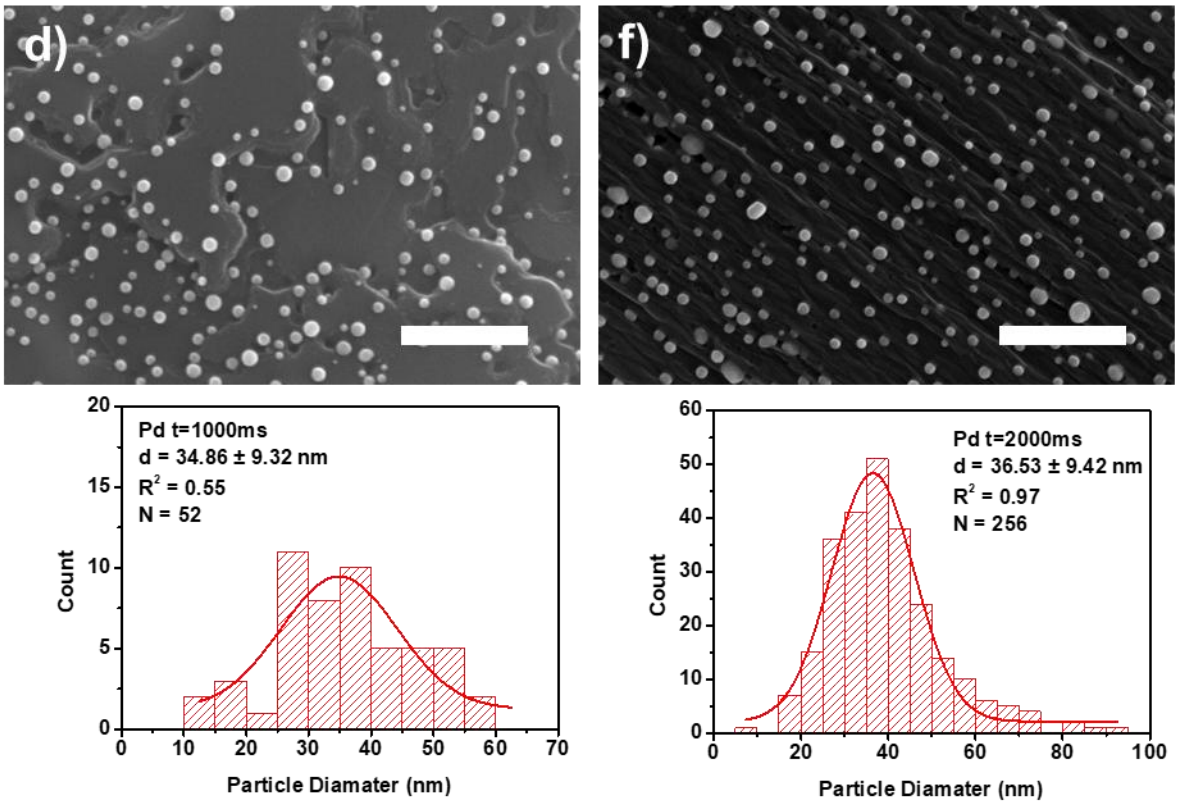

Figure S22. Size distribution of Pd nanoparticles versus flashing time. Scale bar = 500 nm. The flashing time is **a,** 100ms, **b,** 200ms, **c,** 500ms, **d,** 500ms*2, **e,** 1000ms and **f,** 2000ms respectively.

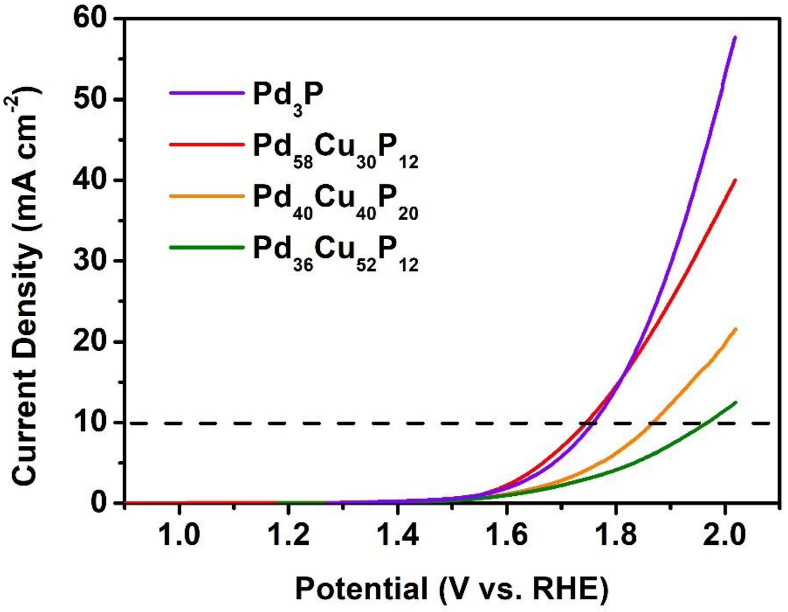

Figure S23. Linear sweeping voltammograms of Pd_3P and Pd-Cu-P alloys.

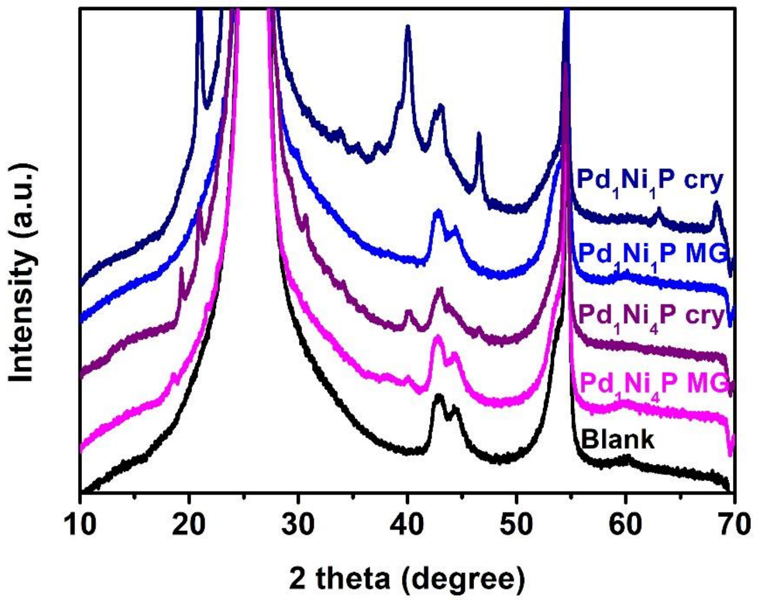

Figure S24. XRD patterns of amorphous and recrystallized Pd_1Ni_3P and Pd_1Cu_1P alloys.

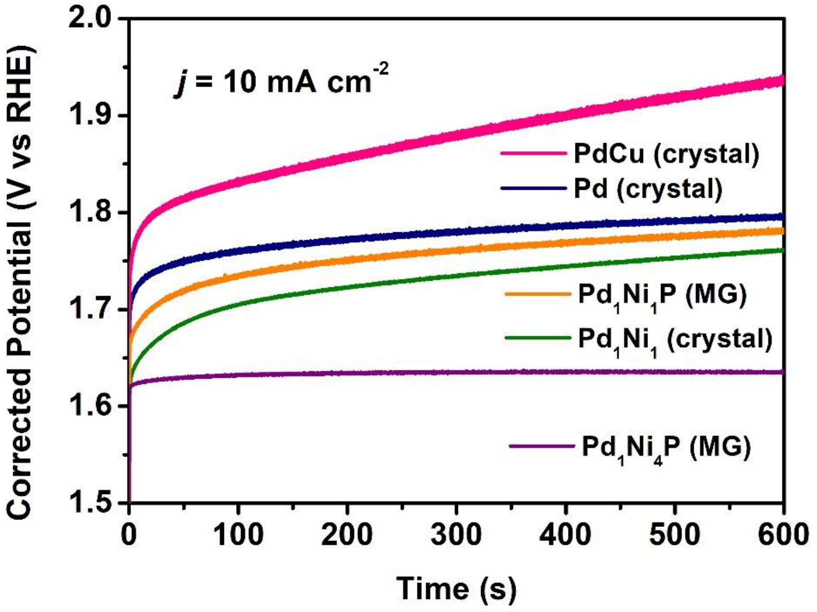

Figure S25. OER stability test result of Pd-Ni-P samples.

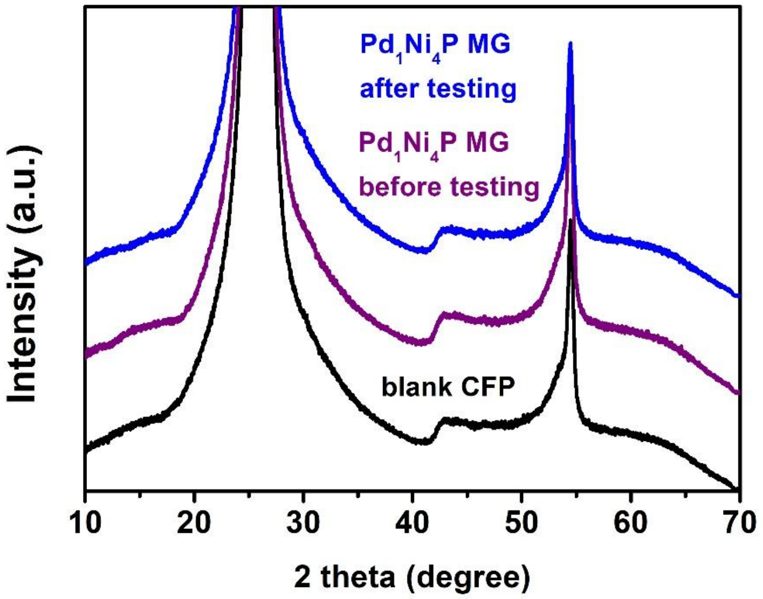

Figure S26. XRD patterns of blank, Pd_1Ni_4P before and after OER stability testing.

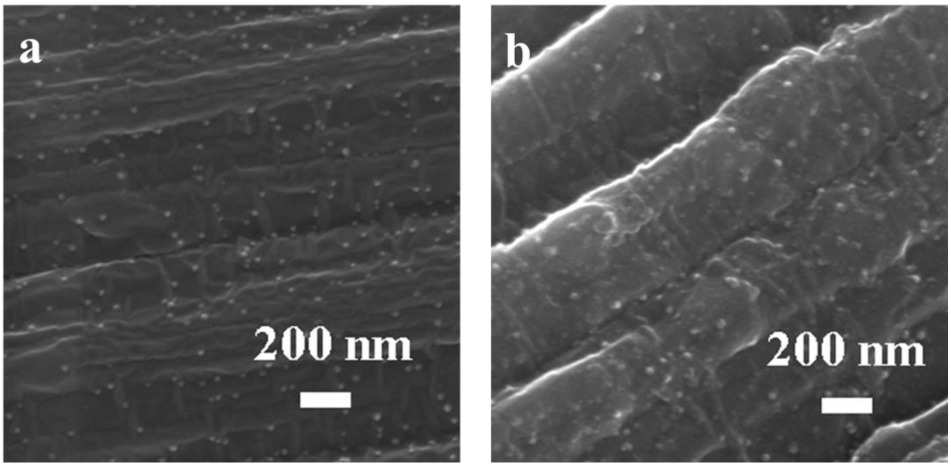

Figure S27. SEM images of Pd_1Ni_4P **a,** before and **b,** after OER stability testing.

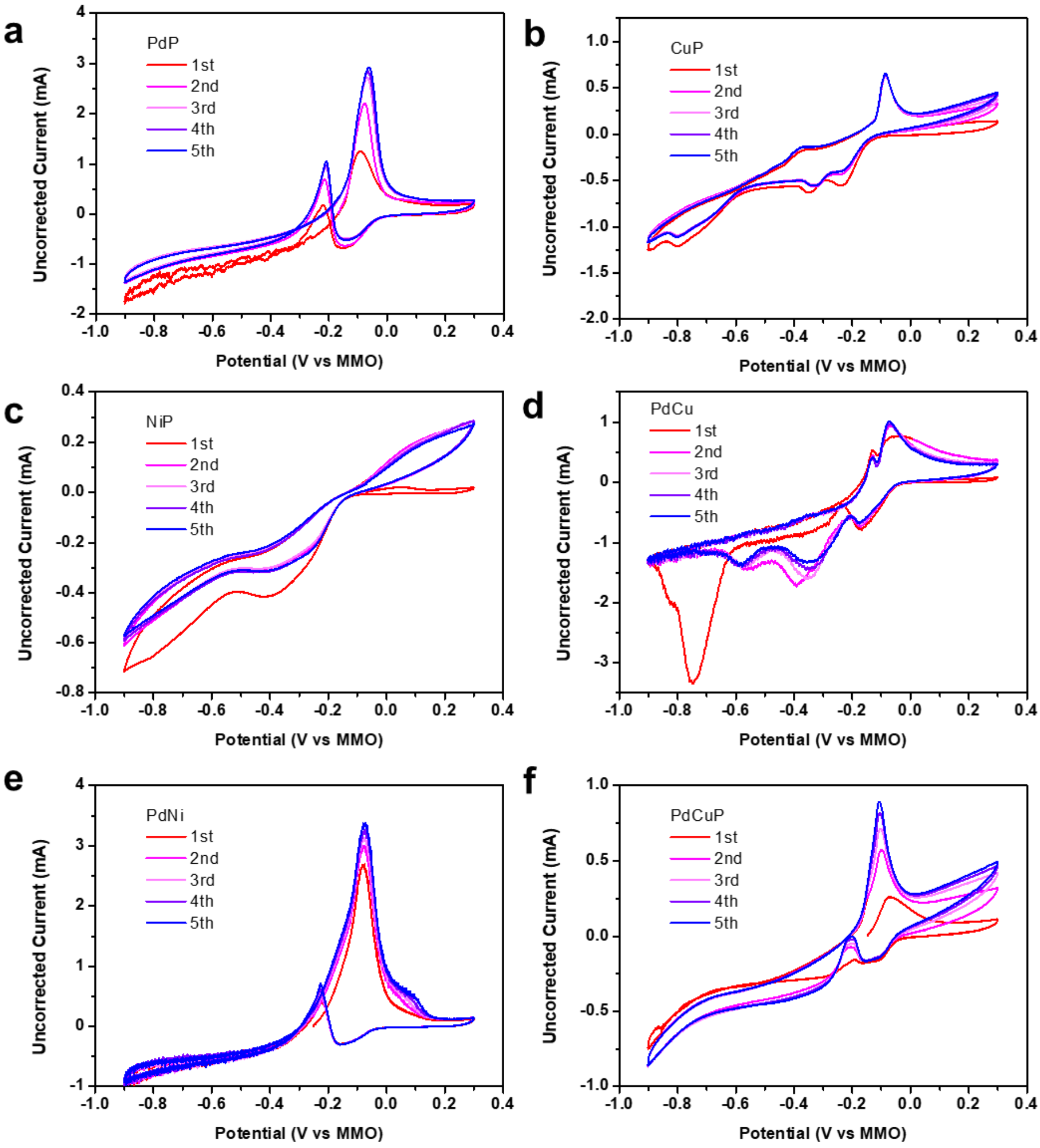
a
PdP
1st
2nd
3rd
4th
5th
Uncorrected Current (mA)
Potential (V vs MMO)
b
CuP
1st
2nd
3rd
4th
5th
Uncorrected Current (mA)
Potential (V vs MMO)
c
NiP
1st
2nd
3rd
4th
5th
Uncorrected Current (mA)
Potential (V vs MMO)
d
PdCu
1st
2nd
3rd
4th
5th
Uncorrected Current (mA)
Potential (V vs MMO)
e
PdNi
1st
2nd
3rd
4th
5th
Uncorrected Current (mA)
Potential (V vs MMO)
f
PdCuP
1st
2nd
3rd
4th
5th
Uncorrected Current (mA)
Potential (V vs MMO)

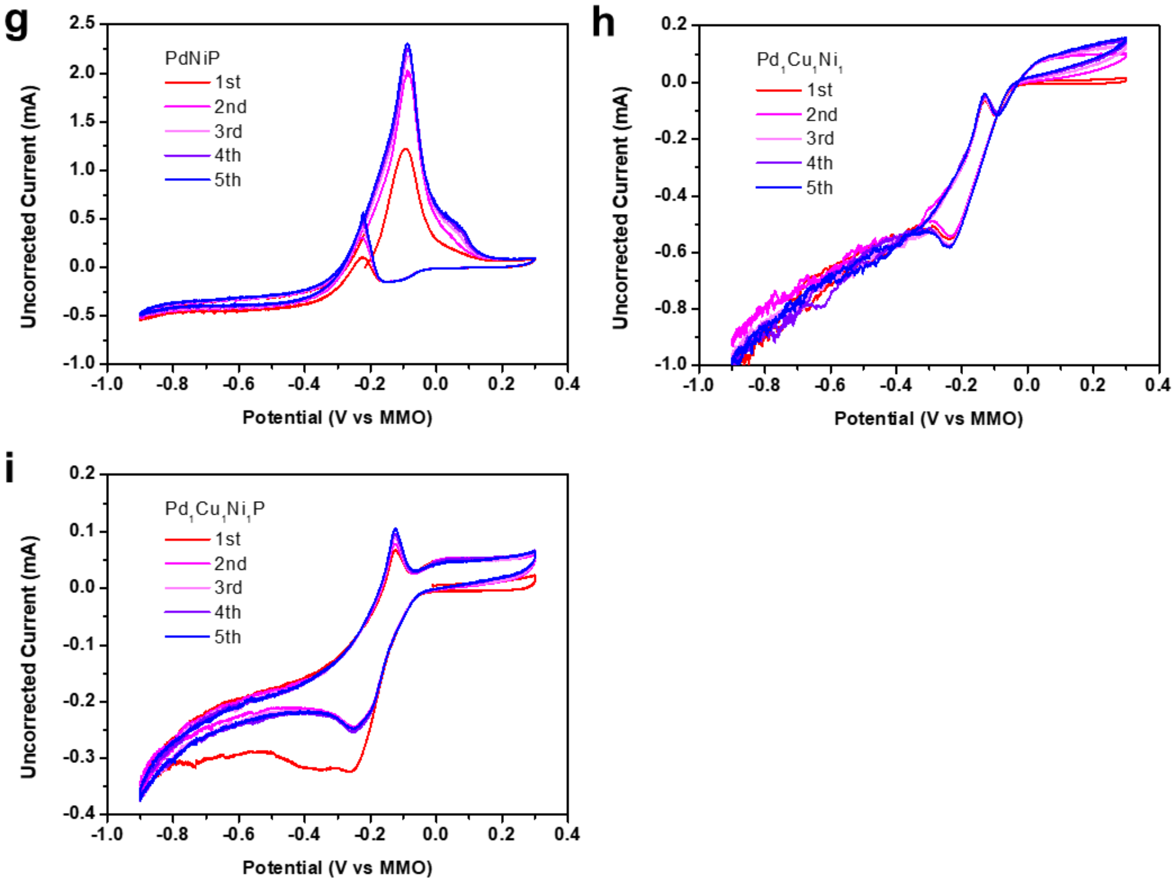

Figure S28. MOR performance of Pd-Ni-P and Pd-Cu-P samples.

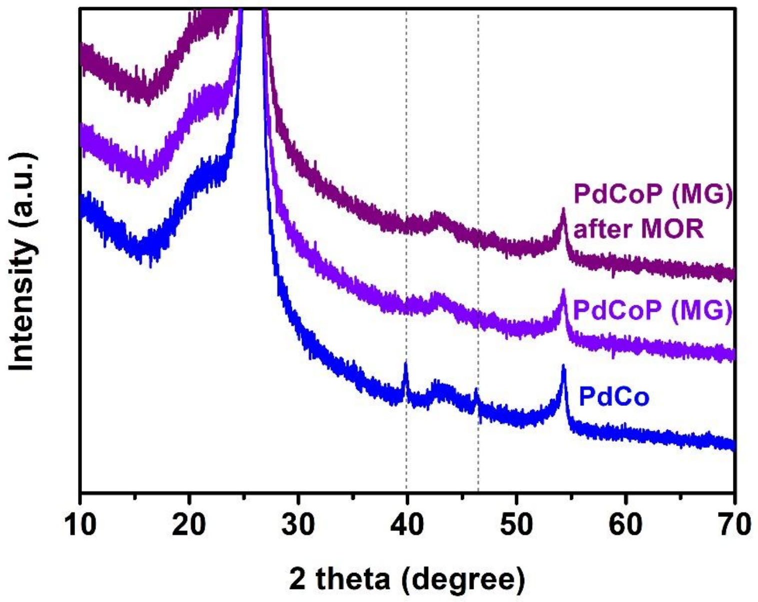

Figure S29. XRD patterns of recrystallized PdCo and amorphous PdCoP alloys.

	Time (ms)	Temperature (K)	Average Ramping Rate From 8.55ms (K/s)
Ramping process	8.55	319.39	NA
	17.52	341.86	2505
	109.04	531.20	2107
	Time (ms)	Temperature (K)	Average Cooling Rate from 1977.34 ms (K/s)
Cooling process	1977.34	1139.00	NA
	1994.00	1114.25	-1485
	2077.34	1014.29	-1182
	Time (ms)	Temperature (K)	Average Cooling Rate from 900K (K/s)
Cooling process	2210.67	900.38	NA
	2595.70	698.23	-525
	2914.34	600.72	-426

Table S1. Ramping and cooling rate calculation

Precursor Ratio (Pd:Ni:P)			FJH time /s	EDX wt% analysis			Product composition
Pd	Ni	P		Pd%	Ni%	P%	
0.5	1.5	0	2	0.38	0.41	NA	$Pd_{34}Ni_{66}$
0.5	1.5	0	2	6.32	7.02	NA	$Pd_{33}Ni_{67}$
1	0	3	0.5	7.45	NA	0.72	$Pd_{75}P_{25}$
1	1	3	0.5	18.22	1.89	1.64	$Pd_{67}Ni_{13}P_{20}$
1	1	3	1	8.56	1.01	0.93	$Pd_{63}Ni_{13}P_{23}$
1	1	3	2	12.39	2.28	1.11	$Pd_{61}Ni_{20}P_{19}$
1	1	3	2	7.85	1.37	0.77	$Pd_{61}Ni_{19}P_{20}$
1.5	0.5	3	2	7.50	0.41	0.72	$Pd_{70}Ni_{7}P_{23}$
1.5	0.5	3	2	5.16	0.34	0.50	$Pd_{69}Ni_{8}P_{23}$
0.5	1.5	3	2	21.85	7.86	1.97	$Pd_{50}Ni_{34}P_{16}$
0.5	1.5	3	2				$Pd_{48}Ni_{38}P_{16}$
0.2	1.8	3	2	4.55	8.2	0.48	$Pd_{22}Ni_{70}P_{8}$
0	1	3	0.5	NA	0.35	NA	Ni
0	1	3	0.5	NA	0.32	0.01	$Ni_{95}P_{5}$

Table S2. EDX elemental analysis of various Pd-Ni-P alloys.

Precursor Ratio (Pd:Cu:P)			FJH time /s	EDX wt% analysis			Product composition
Pd	Ni	P		Pd%	Cu%	P%	
0	1	1	0.5	NA	3.83	0.04	$Cu_{98}P_2$
0	1	1	2	NA	12.51	0.12	$Cu_{98}P_2$
0	1	1	5	NA	8.54	0.04	$Cu_{99}P_1$
0.5	1.5	1	2	8.63	1.06	4.40	$Pd_{20}Cu_{64}P_{16}$
1	1	1	0.5	12.42	8.96	1.49	$Pd_{38}Cu_{46}P_{16}$
1	1	1	2	57.52	35.31	7.17	$Pd_{40}Cu_{41}P_{19}$
1.33	0.66	1	2	71.13	21.44	7.44	$Pd_{54}Cu_{27}P_{19}$
1.33	0.66	1	2	69.36	23.59	7.05	$Pd_{52}Cu_{30}P_{18}$
1.8	0.2	1	2	86.01	6.66	7.33	$Pd_{71}Cu_9P_{20}$
1.8	0.2	1	2	86.78	6.48	6.74	$Pd_{72}Cu_9P_{19}$
1.5	0.5	1	2	78.50	14.41	7.08	$Pd_{62}Cu_{19}P_{19}$
1	0	1	2	13.57	NA	1.31	$Pd_{75}P_{25}$

Table S3. EDX elemental analysis of various Pd-Cu-P alloys.

Movie S1.mp4

Movie S1. Digital video of flash Joule heating process.

Movie S2.mp4

Movie S2. Thermographic video of flash Joule heating process.
